# Acute severe hypoxia induces apoptosis of human pluripotent stem cells by a HIF-1α and P53 independent mechanism

**DOI:** 10.1038/s41598-022-23650-7

**Published:** 2022-11-05

**Authors:** Sofía Mucci, Luciana Isaja, María Soledad Rodríguez-Varela, Sofía Luján Ferriol-Laffouillere, Mariela Marazita, Guillermo Agustín Videla-Richardson, Gustavo Emilio Sevlever, María Elida Scassa, Leonardo Romorini

**Affiliations:** Laboratorios de Investigación Aplicada en Neurociencias (LIAN-CONICET), Fundación Para La Lucha Contra Las Enfermedades Neurológicas de La Infancia (Fleni), Ruta 9, Km 52.5, B1625XAF Belén de Escobar, Provincia de Buenos Aires Argentina

**Keywords:** Biochemistry, Cell biology, Stem cells

## Abstract

Human embryonic and induced pluripotent stem cells are self-renewing pluripotent stem cells (hPSCs) that can differentiate into a wide range of specialized cells. Although moderate hypoxia (5% O_2_) improves hPSC self-renewal, pluripotency, and cell survival, the effect of acute severe hypoxia (1% O_2_) on hPSC viability is still not fully elucidated. In this sense, we explore the consequences of acute hypoxia on hPSC survival by culturing them under acute (maximum of 24 h) physical severe hypoxia (1% O_2_). After 24 h of hypoxia, we observed HIF-1α stabilization concomitant with a decrease in cell viability. We also observed an increase in the apoptotic rate (western blot analysis revealed activation of CASPASE-9, CASPASE-3, and PARP cleavage after hypoxia induction). Besides, siRNA-mediated downregulation of HIF-1α and P53 did not significantly alter hPSC apoptosis induced by hypoxia. Finally, the analysis of BCL-2 family protein expression levels disclosed a shift in the balance between pro- and anti-apoptotic proteins (evidenced by an increase in BAX/MCL-1 ratio) caused by hypoxia. We demonstrated that acute physical hypoxia reduced hPSC survival and triggered apoptosis by a HIF-1α and P53 independent mechanism.

## Introduction

Human pluripotent stem cells (hPSCs), particularly human embryonic and induced pluripotent stem cells (hESCs and hiPSCs, respectively), are self-renewing cells that can differentiate into somatic and germ cell lineages. hESCs are derived from the inner cell mass of human blastocysts, and hiPSCs are reprogrammed from somatic cells by ectopic expression of Yamanaka pluripotency transcription factors OCT-4, SOX-2, KLF-4 and c-MYC^1^. hPSCs are being regarded as potential replacements for tissues in regenerative medicine and are currently used for drug discovery and as models to study human development and diseases^2,3^.

Hypoxia is commonly associated with pathophysiological conditions in which oxygen concentration is inadequate to maintain cellular homeostasis. Nevertheless, embryonic stem cells, in particular, live at low oxygen concentrations (physiologically “normal” hypoxia), which is critical for embryonic and fetal development^4^. Hypoxia can vary in intensity from mild to severe and can be present in acute and chronic forms. The cellular response to oxygen deprivation is governed mainly by a group of oxygen-sensitive transcription factors, named hypoxia-inducible factors (HIF-1α, HIF-2α/EPAS, and HIF-3α). In normoxia, HIF-1α and HIF-2α are polyubiquitinated and targeted for proteasomal degradation. Instead, in low oxygen concentrations, they are stabilized^5^. Once stabilized, dimerize with HIF-1β, which is constitutively expressed, and regulate the transcription of more than 100 genes (e. g. glycolytic enzymes and survival factors) required to cope with low oxygen tensions^6,7^.

Notably, exposure of hPSCs to chronic moderate hypoxia (5% O_2_) induces HIF-2α stabilization, which improves stemness, promotes self-renewal, and protects from spontaneous differentiation^8–10^. This finding is not surprising given that in mammals, while the cardiovascular and hematopoietic systems are not sufficiently developed, the embryonic tissues develop in a hypoxic environment^11^. In contrast, HIF-1α is stabilized in hPSCs after short hypoxic exposure^9^. HIF-1α can prevent cell death, and induce apoptosis or proliferation depending on the cell type and the oxygen concentration^12^. However, the effects of acute severe hypoxia on hPSC viability await to be determined. Acute severe hypoxia may induce hPSC apoptosis as these cells are mitochondrial primed and thought highly sensitive to stressful stimuli^13,14^. In this sense, we and others have reported that chemical compounds routinely used in vitro to mimic hypoxia by HIF-1α stabilization and ROS generation, like cobalt chloride (CoCl_2_) and dimethyloxalylglycine (DMOG)^15–17^, induce apoptosis and necrosis of hPSCs and mouse embryonic stem cells (mESCs)^5,15^.

In the present work, we found that exposure of hPSCs to acute severe (1% O_2_) hypoxia decreased cell viability. Acute 1% O_2_ led to HIF-1α stabilization and the appearance of apoptotic features such as cell ballooning and detachment, increased rate of pyknotic hyperchromatic nuclei, prominent fragmentation of internucleosomal DNA, and activation of initiator CASPASE-9 (mitochondrial apoptosis pathway) and effector CASPASE-3. Notably, siRNA-mediated experiments demonstrated that the increase in cell death was independent of HIF-1α and P53. Finally, the analysis of protein expression levels of relevant BCL-2 family members in hPSCs cultured in 1% O_2_ revealed an increase in BAX/MCL-1 ratio, thereby shifting the balance towards cell death.

## Results

### 1% O_2_ acute hypoxia stabilized HIF-1α in hPSCs

**Figure 1:**
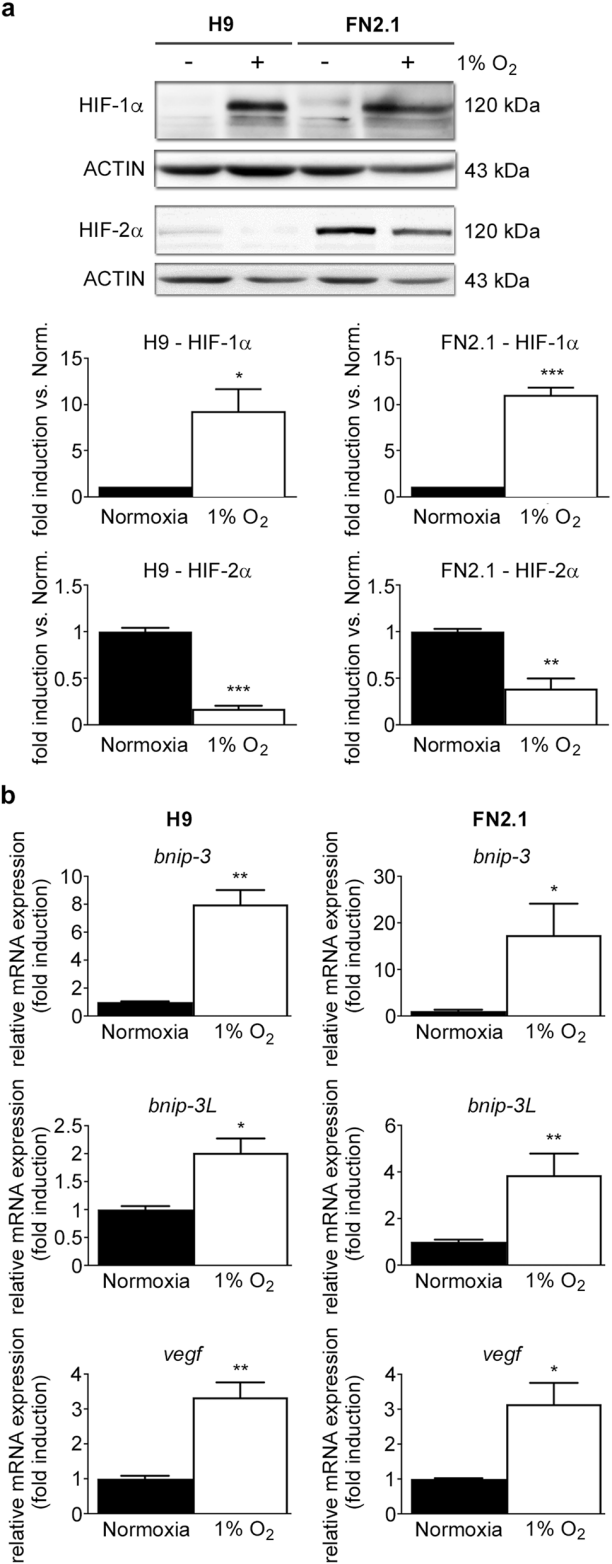
HIF-1α and HIF-2α protein expression levels upon 1% O_2_ incubation. (**a**) *HIF-1α* and *HIF-2α* protein expression levels were quantified by western blot in H9 hESCs and FN2.1 hiPSCs upon 24 h of 1% O_2_ incubation. ACTIN was used as loading control. Representative blots of three independent experiments are shown (full-length or original western blot images are presented in Supplementary Fig. S8 and S9). Bar graphs represent the densitometric quantification of bands. Norm.: normoxia. Data are expressed as means + SEM fold induction relative to normoxia (arbitrarily set as 1) and Statistical analysis was done by Student’s t-test, (*) *p* < 0.05 and (***) *p* < 0.001 versus normoxia. (**b**) Analysis of mRNA expression levels of *BNIP-3*, *BNIP-3L,* and *VEGF* quantified by RT-qPCR in FN2.1 and H9 hPSCs at 24 h post 1% O_2_ treatment. *RPL7* mRNA expression levels were used as normalizer. Graphs show mean + SEM mRNA fold induction relative to normoxia control cells (arbitrarily set as 1) of at least three independent experiments. Statistical analysis was done by Student’s t-test, (*) *p* < 0.05, (**) *p* < 0.01 and (***) *p* < 0.001 versus normoxia.

1% O_2_ severe cellular hypoxia was induced in H9 hESCs and FN2.1 hiPSCs grown on Vitronectin-coated cell culture dishes with fully defined Essential E8 medium (E8) by using a hypoxia incubation chamber. Acute 1% O_2_ hypoxia treatment for 24 h increased *HIF-1α* protein expression levels, which were analyzed by western blot (Fig. 1a). Previous reports determined that *HIF-2α* protein levels are stabilized in hPSCs after long-term (14 days) moderate hypoxia exposure (5% O_2_)^9,18^. Unexpectedly, we found that *HIF-2α* protein expression levels were significantly reduced after incubation at 1% O_2_ for 24 h (Fig. 1a). Besides, mRNA expression levels of *BNIP-3*, *BNIP-3L,* and *VEGF*, well-known transcriptional targets of the HIF-1α/HIF-1β complex^19^, were quantified by RT-qPCR to validate the hypoxic environment (Fig. 1b).

### 1% O_2_ hypoxia triggers apoptosis of hPSCs

**Figure 2:**
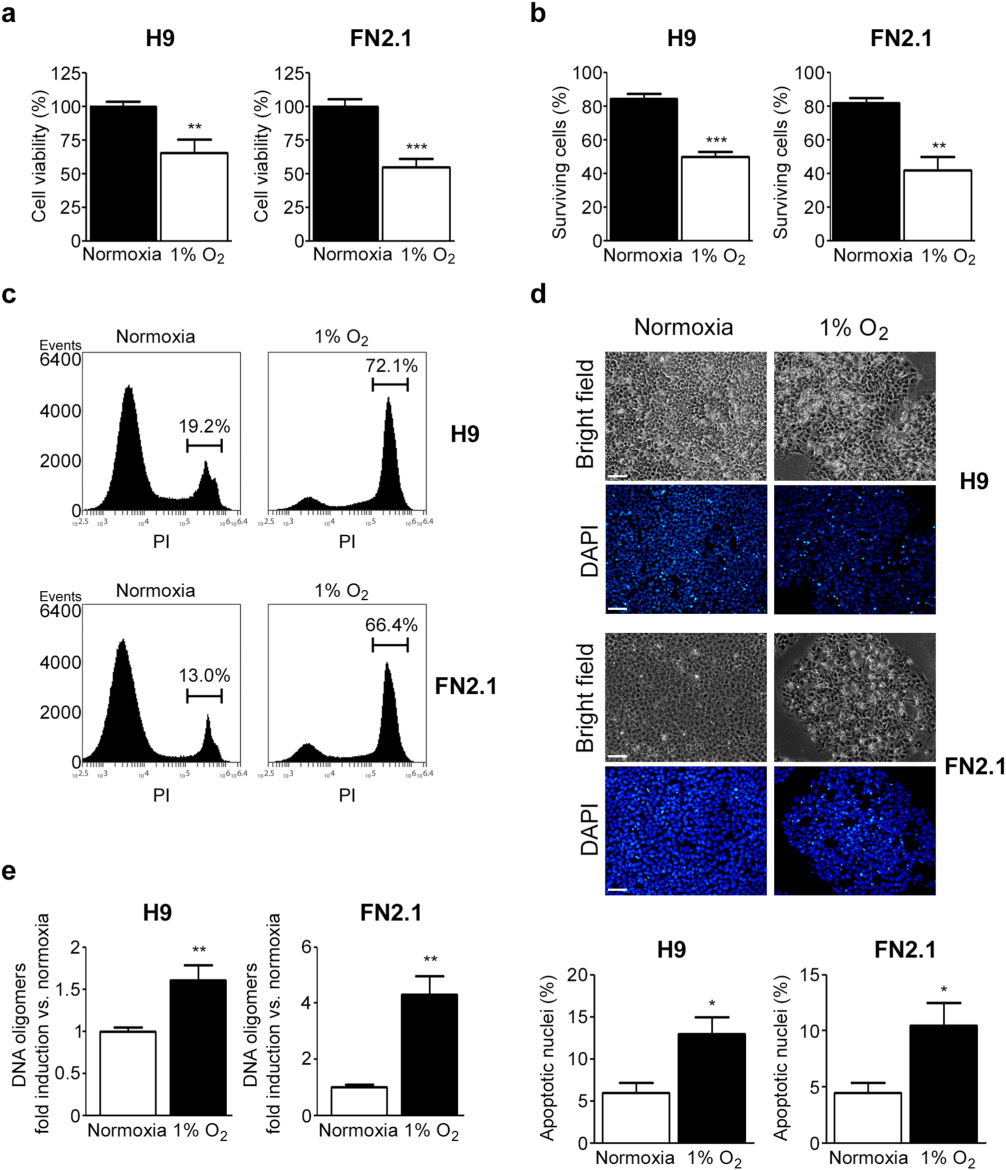
Changes in cell viability and cell death induced by 1% O_2_ treatment in hPSCs. (**a**) H9 and FN2.1 cell viability was analyzed 24 h post 1% O_2_ treatment by XTT colorimetric assay. Mean + SEM from three independent experiments are shown. Statistical analysis was done by Student’s t-test, (**) *p* < 0.01 and (***) *p* < 0.001 versus normoxia. (**b**) Bar graphs show the percentage of surviving cells assessed by Trypan blue exclusion method 24 h after 1% O_2_ incubation. Mean + SEM from at least three independent experiments are shown. Statistical analysis was done by Student’s t-test, (**) *p* < 0.01 and (***) *p* < 0.001 versus normoxia. (**c**) Representative histograms of three independent experiments of Propidium iodide (PI) stained H9 and FN2.1 unfixed cells treated for 24 h with 1% O_2_. The percentage of PI-positive cells (late apoptotic or necrotic) was determined by flow cytometric analysis. (**d**) Chromatin condensation was analyzed by DAPI staining 24 h after 1% O_2_. Figure shows representative images and means + SEM from three independent experiments is graphed for % of apoptotic nuclei. The scale bar represents 100 μm. Statistical analysis was done by one-way ANOVAs followed by Student’s t-test, (*) *p* < 0.05 vs. normoxia. (**e**) Genomic DNA fragmentation into oligomers of 180–200 bp or multiples of that was quantified in H9 and FN2.1 cells at 24 h post 1% O_2_ hypoxia induction using a specific ELISA kit. Mean + SEM fold induction relative to normoxia of three independent experiments is shown. Statistical analysis was done by Student’s t-test, (**) *p* < 0.01 versus normoxia.

We next studied the effects of acute 1% O_2_ hypoxia in hPSC survival. We first quantified the percentage of cell viability after 24 h of 1% O_2_ hypoxia incubation using an XTT/PMS vital dye assay. Importantly, we found that under the mentioned experimental conditions, the cell viability rate significantly decreased in both H9 hESCs and FN2.1 hiPSCs (Fig. 2a). Similar results were observed when live and dead cells were counted using Trypan blue dye (Fig. 2b). Additionally, cell death was determined by propidium iodide (PI) staining and flow cytometry analysis. Figure 2c and Supplementary Figure S1 show that the percentage of PI-positive hPSCs, due to loss of plasma membrane integrity (an event that occurs in late apoptosis or necrosis), increased upon acute hypoxia treatment (1% O_2_, 24 h).

Next, to gain insight into the mechanism of cell death triggered by acute 1% O_2_ hypoxia, we evaluated the appearance of apoptotic features in hESCs (H9) and hiPSCs (FN2.1). As some of the criteria used to identify apoptotic cells are chromatin condensation paralleled by cellular ballooning and detachment, we assessed these morphological changes by DAPI staining of nuclear DNA and bright-field microscopic images of hPSCs under normoxia or hypoxia (1% O_2_), respectively. We found that 1% O_2_ hypoxia treatment for 24 h induced both the percentage of pyknotic apoptotic nuclei (increased DAPI brightness due to chromatin condensation) and the presence of ballooned and detached cells (Fig. 2d).

Further, we measured DNA fragmentation (cytoplasmic oligonucleosomal fragments of inter-nucleosomal cleavage of DNA), a late event in the apoptotic cascade. Figure 2e shows a significant induction in the proportion of DNA oligomers, quantified with an ELISA-based immunoassay, after acute hypoxia treatment (1% O_2_, 24 h) in both H9 and FN2.1 cell lines.

**Figure 3:**
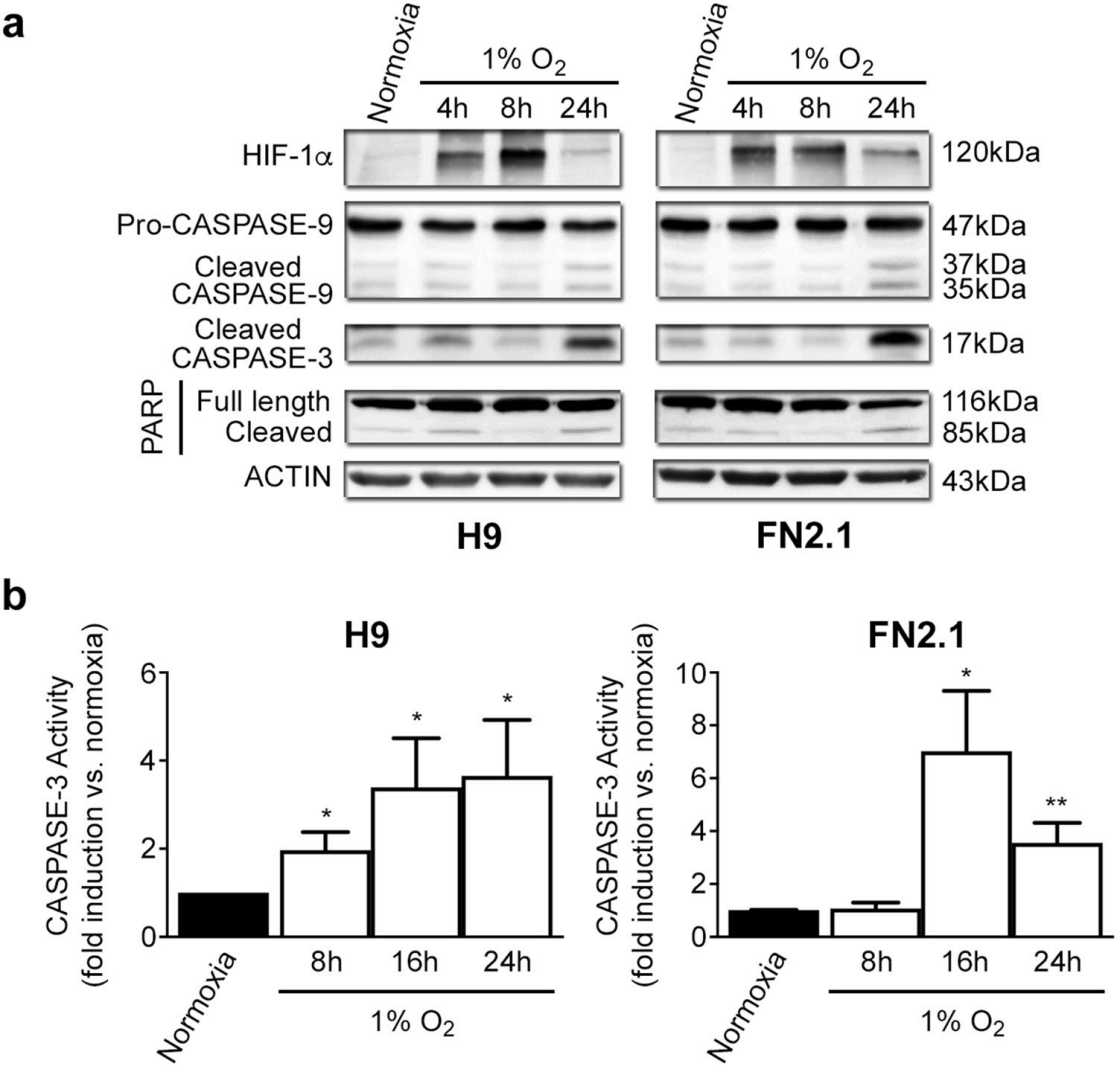
CASPASE-9, CASPASE-3 activation, and PARP cleavage upon 1% O_2_ hypoxia induction. (**a**) Cleavage and activation of initiator CASPASE-9, effector CASPASE-3, PARP proteolysis (CASPASE-3 substrate), and HIF-1α stabilization were analyzed by western blot in H9 hESCs and FN2.1 hiPSCs at 4-, 8- and 24-h post 1% O_2_ incubation. ACTIN was used as loading control. Representative blots of three independent experiments are shown (original images are presented in Supplementary Fig. S10 and S11). (**b**) CASPASE-3 activity was determined upon 8-, 16- and 24-h of 1% O_2_ treatment by measuring the proteolysis of CASPASE-3 substrate Z-DEVD-R110. Specific activity was calculated as fluorescence/number of cells and expressed as fold induction against normoxia. Mean + SEM fold induction relative to normoxia (arbitrarily set as 1) of three independent experiments is shown. Statistical analysis was done by Student’s t-test, (*) *p* < 0.05 and (**) *p* < 0.01 versus normoxia.

Another relevant criterion used to determine that cells die by apoptosis is the activation of caspases (initiator and effector). In this sense, we found by western blot analysis that initiator PRO-CASPASE-9 (47 kDa) was cleaved into active fragments (37/35 kDa) upon acute severe hypoxia (1% O_2_) treatment in hPSCs (Fig. 3a and Supplementary Fig. S2). Moreover, the detection of cleaved effector CASPASE-3 (appearance of p17 fragment) exposed a time-dependent activation of CASPASE-3 mediated by 1% O_2_ treatment, which was accompanied by HIF1-α stabilization and CASPASE-9 cleavage. CASPASE-3 activation was also confirmed by immunofluorescent staining of the p17 fragment (Supplementary Fig. S3) and by measuring the fluorescence generated by proteolysis of the fluorogenic CASPASE-3 substrate Z-DEVD-R110 (Fig. 3b). Besides, time course studies showed the presence of cleaved PARP, a well-known target of CASPASE-3 (Fig. 3a and Supplementary Fig. S2). Interestingly, acute severe hypoxia (1% O_2_) resulted in the activation of initiator and effector caspases which display very similar kinetics in both cell types (H9 hESCs and FN2.1 hiPSCs). Importantly, results demonstrate that acute severe hypoxia induces apoptosis in hPSCs. Moreover, due to CASPASE-9 cleavage, we concluded that the mitochondrial-mediated apoptosis pathway participates in this event.

### Involvement of HIF-1α in 1% O_2_-induced apoptosis in hPSCs

**Figure 4:**
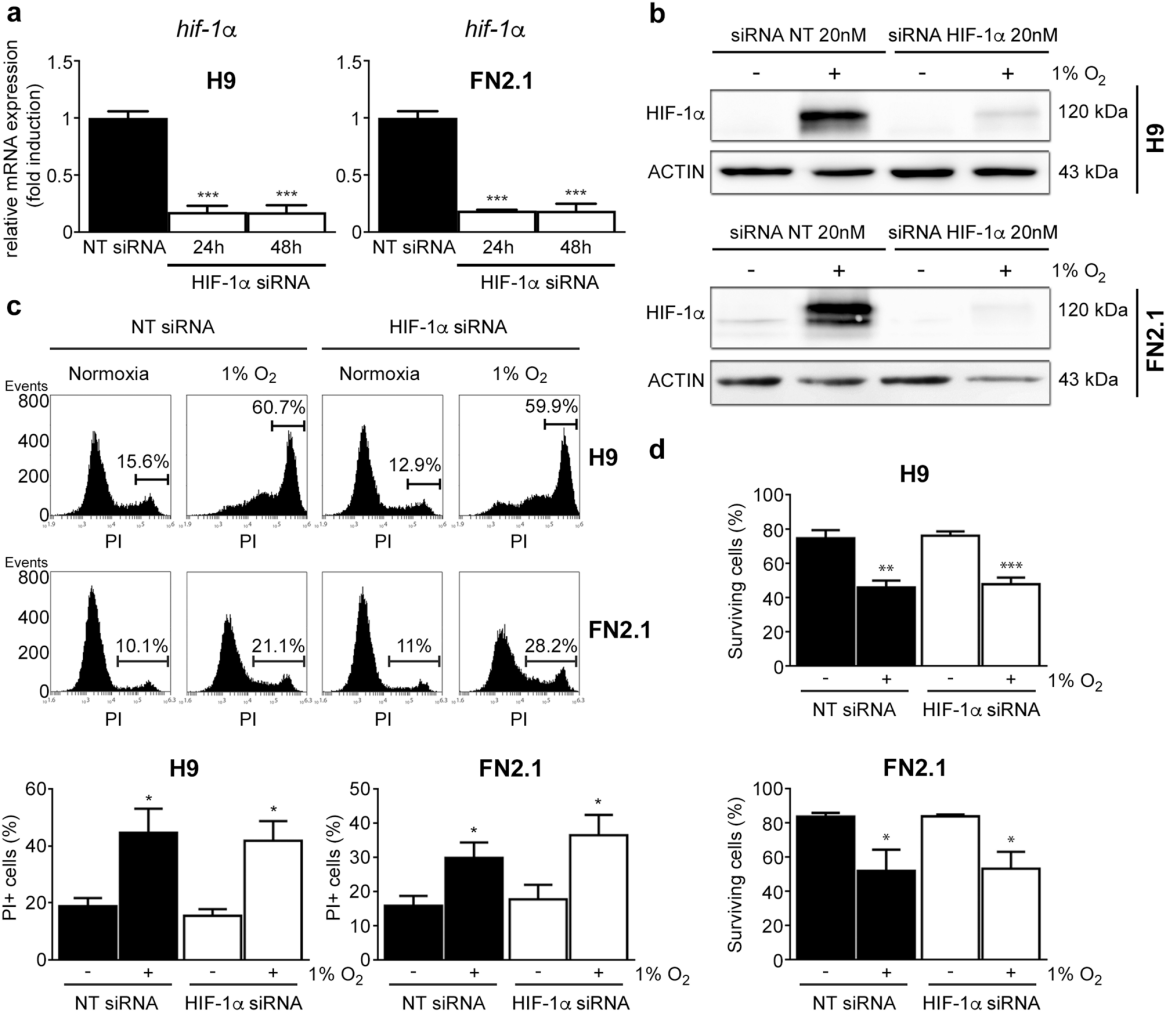
Effect of siRNA-mediated down regulation of HIF-1α in hPSCs cell viability and death upon 1% O_2_ hypoxia induction. H9 hESCs and FN2.1 hiPSCs were transfected with negative control non-targeting siRNA (NT siRNA) (20 nM) or HIF-1α siRNA (20 nM) and then: (**a**) mRNA expression levels of *HIF-1α* were analyzed by RT-qPCR at 24 and 48 h post siRNAs transfection. *RPL7* expression was used as normalizer. Graph shows mean + SEM mRNA fold induction relative to NT siRNA transfectants arbitrarily set as 1 from three independent experiments. Statistical analysis was done by Student’s t-test, (***) *p* < 0.001 versus NT siRNA. (**b**) Expression levels of HIF-1α were analyzed by western blot in H9 and FN2.1 cells at 48 h post siRNAs transfection. HIF-1α was stabilized by hypoxia (1% O_2_ for 24 h starting at 24 h post siRNAs transfection) treatment. ACTIN was used as loading control. Representative blots are shown (full-length images are presented in Supplementary Fig. S12). (**c**) Representative histograms of Propidium iodide (PI) stained H9 and FN2.1 unfixed cells at 48 h post siRNA transfection. 24 h 1% O_2_ hypoxia treatment was started at 24 h post siRNA transfection. The percentage of PI-positive cells (late apoptotic or necrotic) was determined by flow cytometric analysis. Mean + SEM from three independent experiments are graphed. Statistical analysis was done by Student’s t-test, (*) *p* < 0.05 vs. normoxia. (**d**) Histograms show the percentage of surviving cells assessed by Trypan blue exclusion method at 48 h post siRNA transfection. 24 h after transfection cells were incubated for 24 h at 1% O_2_. Mean + SEM from three independent experiments is shown. Statistical analysis was done by Student’s t-test, (*) *p* < 0.05, (**) *p* < 0.01, and (***) *p* < 0.001 vs. NT siRNA.

Next, to test the involvement of HIF-1α in hypoxia-induced apoptosis in hPSCs, we used specific siRNA to downregulate *HIF-1α* expression. The efficiency of siRNA-knockdown was monitored by RT-qPCR and western blot in hESCs (H9) and hiPSCs (FN2.1) cultured in defined E8 medium and transfected with either non-targeting control siRNA (NT siRNA) or specific siRNA. We observed that siRNA transfection led to a significant decrease in *HIF-1α* mRNA and protein expression levels (Fig. 4a,b and Supplementary Fig. S4). Interestingly, we found that in hPSCs siRNA-mediated downregulation of HIF-1α did not revert the increased apoptosis or necrosis induced by 1% O_2_ hypoxia as judged by PI staining and Trypan blue dye exclusion data (Fig. 4c a,d). Taken together, the above results suggest that acute severe hypoxia induces hPSC cell death by a HIF-1α independent mechanism.

### Apoptotic protein profiling in 1% O_2_ hypoxia-treated hPSCs

hPSCs are very susceptible to undergo apoptosis due to their higher state of mitochondrial priming, a lowered cell intrinsic threshold for initiating mitochondrial-induced apoptosis, based on the balance between pro- and anti-apoptotic protein members of the BCL-2 family^5,14,20^. Bearing this in mind, we performed western blot assays to analyze the expression levels of crucial BCL-2 family members in hPSCs at 4-, 8- and 24-h post-hypoxia (1% O_2_).

**Figure 5:**
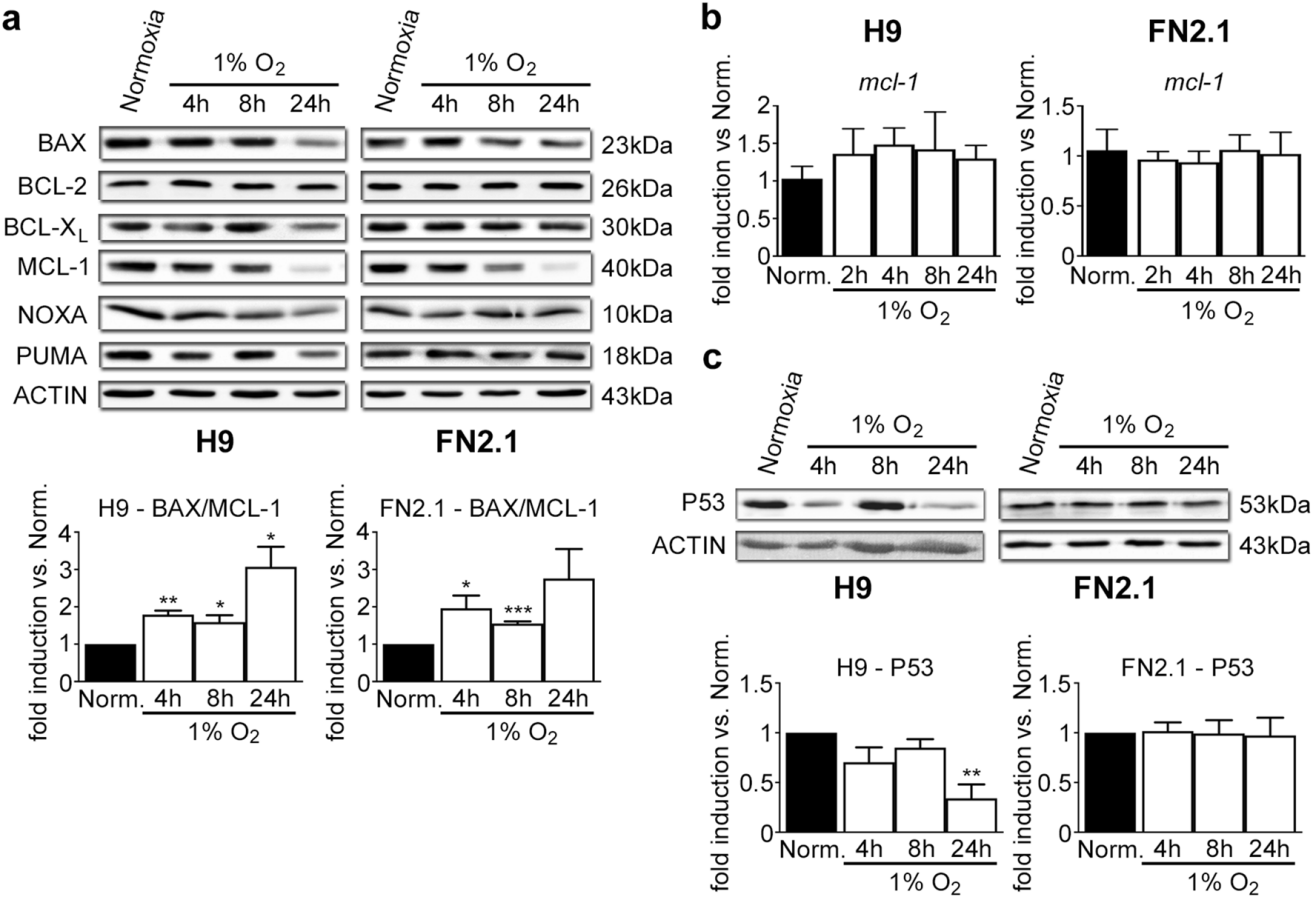
BCL-2 family members and P53 protein expression levels. Expression levels of (**a, c**) BCL-2 family members, including BCL-X_L_ (anti-apoptotic), BCL-2 (anti-apoptotic), MCL-1 (anti-apoptotic), BAX (pro-apoptotic), NOXA (pro-apoptotic) and PUMA (pro-apoptotic) (**a**) or P53 (**c**) were analyzed by western blot in H9 and FN2.1 cells at 4, 8 and 24 h upon 1% O_2_ hypoxia treatment. ACTIN was used as loading control. Representative blots of three independent experiments are shown (original western blot images are presented in Supplementary Fig. S13, S14, and S15). Bar graphs represent the densitometric quantification of bands. Data are expressed as means + SEM fold induction relative to normoxia (Norm.) and statistical analysis was done by Student’s t-test, (*) *p* < 0.05, (**) *p* < 0.01, and (***) *p* < 0.001 versus normoxia. (**b**) mRNA expression levels of *MCL-1* were analyzed by RT-qPCR at 2, 4, 8, and 24 h upon 1% O_2_ hypoxia induction. *RPL7* expression was used as normalizer. Graph shows mean + SEM mRNA fold induction relative to normoxia (arbitrarily set as 1) from three independent experiments. Statistical analysis was done by Student’s t-test.

Particularly, we studied the expression levels of BCL-X_L_ and BCL-2, key anti-apoptotic proteins; BAX, pro-apoptotic protein constitutively active at the Golgi in hPSCs^14^; and MCL-1 (anti-apoptotic), PUMA (pro-apoptotic) and NOXA (pro-apoptotic) which are highly expressed and rapidly responding proteins in hPSCs^21,22^. Results shown in Fig. 5a and Supplementary Figure S5 indicate that BCL-X_L_, BCL-2, BAX, PUMA, and NOXA expression levels did not significantly change upon hypoxia treatment at early time points. However, MCL-1 expression levels significantly decreased as soon as 4 h post-1% O_2_ incubation in both H9 hESCs and FN2.1 hiPSCs. Nevertheless, *MCL-1* mRNA expression levels (quantified with RT-qPCR) were not altered with 1% O_2_ treatment (Fig. 5b). In addition, we did not detect changes in the mRNAs expression levels of the rest of the BCL-2 family genes except for those of NOXA that significantly increased in FN2.1 hiPSCs upon 24 h of 1% O_2_ treatment (Supplementary Fig. S6).

We further calculated the ratio of the protein expression levels of BAX to MCL-1 and compared them between normoxia and different time points upon hypoxia (1% O_2_) induction. We found that BAX/MCL-1 ratio was increased by hypoxia (H9: 1.79 ± 0.11, 1.59 ± 0.18 and 3.07 ± 0.54-fold induction vs. normoxia for 4 h, 8 h, and 24 h 1% O_2_ hypoxia, respectively; FN2.1: 1.96 ± 0.34, 1.55 ± 0.06 and 2.76 ± 0.79-fold induction vs. normoxia for 4 h, 8 h and 24 h 1% O_2_ hypoxia, respectively) (Fig. 5a). These findings suggest that BAX/MCL-1 ratio may serve as a rheostat to determine the susceptibility of hPSCs to undergo apoptosis upon hypoxia (1% O_2_) exposure.

On the other hand, P53 is a transcription factor known to be activated by hypoxia^23^. P53 can induce genes that codify for some proteins involved in the intrinsic apoptosis pathway^24^. To study the possible role of P53 in our acute hypoxia model, we first quantified P53 expression levels by western blot in hPSCs incubated for 4, 8, and 24 h under 1% O_2_. We found that P53 expression levels were not upregulated by 1% O_2_ in hPSCs (Fig. 5c). P53 expression levels significantly decreased in H9 hESCs upon 24 h of 1% O_2_ treatment (Fig. 5c). Moreover, siRNA-mediated downregulation of P53 did not revert the increased apoptosis or necrosis induced by 1% O_2_ as judged by PI staining and Trypan blue dye exclusion data (Supplementary Fig. S7). These results discard P53 participation in 1% O_2_-induced apoptosis in hPSCs.

### MCL-1 involvement in 1% O_2_ hypoxia-mediated hPSC cell death

**Figure 6:**
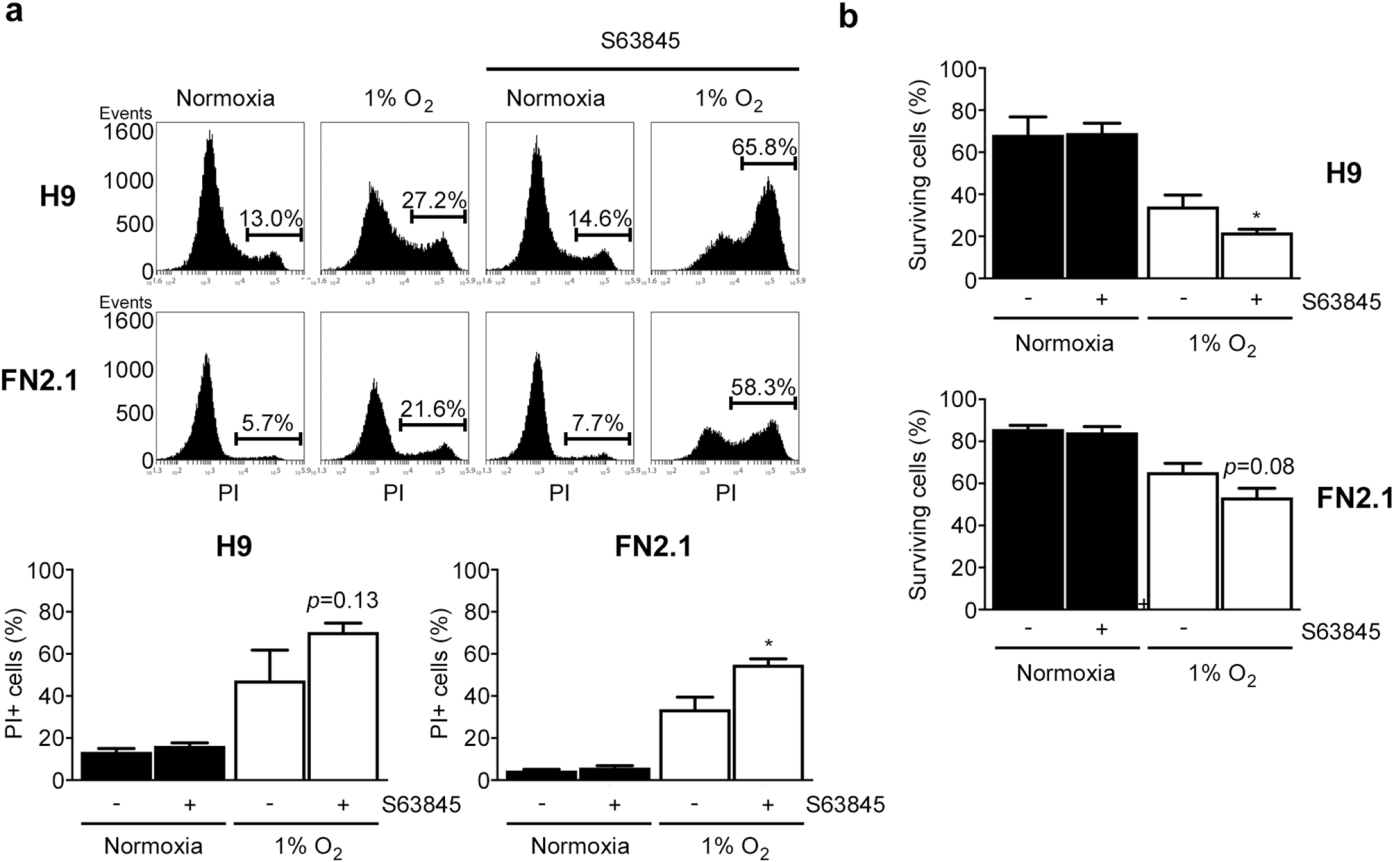
MCL-1 involvement in 1% O_2_ hypoxia-mediated hPSCs cell death*.* (**a**) Representative histograms of Propidium iodide (PI) stained H9 and FN2.1 unfixed cells treated for 24 h with 1% O_2_ in the presence or not of MCL-1 inhibitor S63845 (50 nM). The percentage of PI-positive cells (late apoptotic or necrotic) was determined by flow cytometric analysis. Mean + SEM from three independent experiments is graphed. Statistical analysis was done by Student’s t-test, (*) *p* < 0.05 vs. normoxia. (**b**) Bar graphs show the percentage of H9 and FN2.1 surviving cells assessed by Trypan blue exclusion method 24 h after 1% O_2_ incubation in the presence or not of MCL-1 inhibitor S63845 (50 nM). Mean + SEM from three independent experiments is shown. Statistical analysis was done by Student’s t-test, (**) *p* < 0.01 and (***) *p* < 0.001 versus normoxia.

Finally, to test whether MCL-1 could be involved in 1% O_2_-mediated apoptosis of hPSCs, we evaluated the effect of the highly selective MCL-1 inhibitor S63845 (a small molecule that specifically binds with high affinity to the BH3-binding groove)^25^ on cell death induced by 1% O_2_ treatment. Interestingly, MCL-1 inhibition enhanced late apoptosis or necrosis rate (by flow cytometry analysis with PI staining) triggered by 24 h of 1% O_2_ incubation in both H9 and FN2.1 cells (Fig. 6a). Similar results were obtained when viable cells were quantified using Trypan blue dye-exclusion assay. As shown in Fig. 6b, the percentage of surviving cells that markedly decreased upon 24 h of 1% O_2_ treatment was even lower by MCL-1 inhibition in hPSCs. These results suggest that MCL-1 could exert a protective role in hPSC survival and that its downregulation sensitizes hPSCs to 1% O_2_-induced apoptosis.

## Discussion

Low levels of O_2_ occur naturally in developing embryos. Moreover, it has become evident that embryonic stem cells frequently occupy hypoxic niches and low O_2_ levels (generally < 5% O_2_) regulate their differentiation^4^. In this regard, several reports indicate that the culture of hPSCs at 2–5% O_2_ is advantageous for their maintenance in terms of reduced spontaneous differentiation, improved proliferation, and increased expression of crucial pluripotent markers^8,18,26–29^.

Contrarily, in the absence of O_2_ or severe hypoxia, most cell types undergo cell death. However, the mechanisms triggered by hPSCs exposed to severe hypoxia remain elusive. For this reason, in the present study, we explored the effects caused by 1% O_2_ exposure in hESCs and hiPSCs. Often severe hypoxia causes HIF-1α and HIF-2α stabilization. We observed that exposure of hPSCs to acute (less than 24 h) severe (1% O_2_) hypoxia caused HIF-1α stabilization. Surprisingly, upon 1% O_2_, the expression levels of HIF-2α decreased significantly. Interestingly, this particular HIF-2α behavior upon hypoxia incubation was only previously reported in a few cellular contexts^30,31^. These researchers demonstrated that HIF-2α protein expression levels were downregulated by hypoxia-induced CALPAIN activation^30^ or by an unknown O_2_-independent mechanism^31^. Nevertheless, the mechanism by which HIF-2α expression levels decreased upon 1% O_2_ treatment in hPSCs awaits to be determined.

According to a specific context and cell type, activated HIF-1α can lead to the regulation of more than 100 genes, some of them involved in cell protection or cell death. In consequence, depending on the cellular context, HIF-1α can prevent cell death, induce apoptosis or even stimulate cell proliferation^12^. Interestingly, we found that exposure of hPSCs to acute (less than 24 h) severe (1% O_2_) hypoxia caused a significant decrease in cell viability. The reduction in cell viability encompassed cellular ballooning and detachment, CASPASE-9 and CASPASE-3 activation, PARP cleavage, pyknotic nuclei, and DNA fragmentation indicative of apoptotic processes.

HIF-1α can regulate apoptosis by transcriptional regulation of pro- or anti-apoptotic proteins like BNIP-3 and BNIP-3L^19,32,33^, BCL-2^34^, BCL-X_L_^35^, BAX^36^ and NOXA^37^ or by P53 stabilization^38^. We demonstrated that 1% O_2_ significantly induced mRNA levels of *BNIP-3* and *BNIP-3L* in both hESCs and hiPSCs and *NOXA* only in FN2.1 hiPSCs. We even found that siRNA-mediated downregulation of HIF-1α did not significantly affect the extent of cell death triggered by low O_2_ levels in hPSCs, suggesting the participation of alternative pathways.

It is noteworthy that P53 regulates alternative pathways involved in programmed cell death, so we explored the participation of this tumor suppressor in hypoxia-mediated apoptosis. We found that P53 levels remained unaltered (FN2.1 hiPSCs) or significantly decreased (H9 hESCs) in hPSCs exposed to 1% O_2_ for 24 h, and its siRNA-mediated downregulation did not affect the rate of cell death, discarding P53 participation in this event. In this regard, there is controversy about whether and to what extent P53 is stabilized and activated under reduced O_2_ tensions. On the one hand, An et al*.* claimed that P53 is stabilized by HIF-1α^39^. On the other hand, Wang et al*.* challenged this mechanism by showing that P53 accumulates only under extremely severe hypoxia (0.02% O_2_) with the ataxia-telangiectasia mutated and RAD3-related protein kinase contributing to this stabilization^40^. Besides, it is also worth mentioning that P53 is a labile protein with a short half-life^41^, thus suggesting that the observed lack of increase or even decrease in the *P53* protein expression levels might be in line with the P53 hypoxia-induced apoptosis independence.

The intrinsic mitochondrial pathway induces apoptosis in response to a wide variety of stimuli or cellular stresses such as hyperthermia, hypoxia, DNA damage, excessive free radicals, or toxins, among others. Although diverse in nature, these stimuli lead to the permeabilization of the outer mitochondrial membrane, mediated by the BCL-2 family members. The presence of up to four conserved motifs known as BCL-2 homology domains (BH1–BH4) characterizes this family of proteins. In addition to BCL-2, several other proteins BCL-X_L_, BCL-W, and MCL-1, have an anti-apoptotic effect. On the other hand, the members of the pro-apoptotic group are divided into two subgroups: the BAX-subfamily consisting mainly of BAX and BAK, which contain BH1, BH2, and BH3 domains, and the members of the BH3-only subfamily, which possess only the BH3 motif, like BAD, NOXA, PUMA, etc.^42^. The fate of a cell resides in the balance between the levels of pro- and anti-apoptotic BCL-2 proteins, which the prevailing cellular signaling modulates to tip the equilibrium towards survival or death. Bearing this in mind, we thought to determine if hypoxia induces changes in the levels of key BCL-2 factors such as BCL-2, BCL-X_L_, MCL-1, BAX, PUMA, and NOXA displayed by hPSCs. After 24 h in 1% O_2_, only the levels of anti-apoptotic MCL-1 decreased at early time points (4 and 8 h), while the abundance of the remaining analyzed factors did not vary significantly. The finding that MCL-1 levels are modulated by O_2_ concentration in vitro is in line with previous studies, although whether MCL-1 is up or downregulated may be cell type and O_2_ concentration dependent^19,43,44^.

Interestingly, MCL-1 is unique among pro-survival BCL-2 family members in that it is essential for embryonic development^45^, and is crucial to the survival of multiple cellular lineages. Besides, Rasmussen et al*.* demonstrated that MCL-1 is a fundamental regulator of mitochondrial dynamics in hPSCs, independent of its role in apoptosis^46^. MCL-1 has a very short half-life, suggesting that the control of its expression involves transcriptional and post-translational processes. Moreover, it has been shown that the transcriptional level of *MCL-1* remains unaltered during hypoxia. Accordingly, we did not detect changes in *MCL-1* mRNA expression levels at least at the O_2_ tension and periods tested, even though it has been reported that the human *MCL-1* promoter contains an active hypoxia response element^43^.

It is worth mentioning that during hypoxia, cells activate adaptive mechanisms^47^, which include inhibition of energy-costly mRNA translation to subsist O_2_ deficiency^48–50^. Thus, it is conceivable that a decrease in global translation may suffice to explain MCL-1 downregulation, given that a more dramatic effect would be observed on the levels of labile proteins, such as MCL-1, than in stable proteins. We propose that in severe hypoxia, the ratio of rapidly turned over to long-lived proteins can increase significantly, and relevant cellular processes (e.g., apoptosis) that depend on the balance between short-lived and long-lived proteins will be affected. However, to test this hypothesis, further studies need to be performed.

In line with this hypothesis, recently we demonstrated that Roscovitine, a cyclin-dependent kinase (CDK) inhibitor, triggers apoptosis in hESCs but not in primary fibroblasts. Roscovitine decreases transcription by inhibiting CDK7 and CDK9, which are responsible for the phosphorylation of the carboxy-terminal domain of the RNA polymerase II largest subunit. Importantly, we observed that Roscovitine-induced apoptosis was accompanied by MCL-1 downregulation. *MCL-1* is a crucial transcriptional target of RNA pol II and, as previously mentioned, a short-lived transcript, so we proposed that repression of transcription might be the cause of its relatively rapid elimination. The observation that Roscovitine induces cell death in H9 hESCs but not in human fibroblasts suggests that these pluripotent cells rely more heavily on MCL-1 for their survival than primary fibroblasts, thus emerging as a critical regulator of hESC fate^51^.

However, despite the benefit of pluripotent cells in regenerative medicine, hPSC apoptosis remains an obstacle to its applications in hypoxic environments^52^. Thus, elucidating the mechanisms involved in hypoxia-induced apoptosis may help to control hPSC fate, and minimize cell death within hypoxic niches. To face this obstacle, we recently demonstrated that CoCl_2_, a widely used chemical surrogate of hypoxia, triggered apoptotic cell death in hPSCs via a NOXA-mediated HIF-1α and HIF-2α independent mechanism^5^. Our findings suggest that MCL-1 could regulate hPSC survival after exposure to 1% O_2_ and that depending on the hypoxia inducer, alternative gene expression programs become operative in hPSCs. In line with our findings, it was demonstrated that high expression of NOXA sensitizes hPSCs for rapid cell death^53^. It is then conceivable that the decrease in the expression levels of MCL-1 triggered by 1% O_2_ or the reduction of MCL-1/NOXA complexes due to S63845 treatment may turn hPSCs more susceptible to dye. However, whether MCL-1 is a key factor that sustains hPSC survival at 1% O_2_ remains still an open question and more experiments are needed to confirm this hypothesis.

## Methods

### Cell lines and culture

hESCs line WA09 (H9)^2^ was purchased from WiCell Research Institute (http://www.wicell.org) and hiPSCs line FN2.1 was previously derived from human foreskin fibroblasts^54^. All methods were performed by the relevant guidelines and regulations. Ethical approval was received by the local Ethics Committee (Comité de ética en Investigaciones biomédicas del Instituto FLENI) and written informed consent was obtained from the donor before foreskin fibroblast isolation. hPSCs were cultured on Vitronectin (VTN-N, Gibco) (0.5 µg/cm^2^) coated dishes in combination with fully defined Essential 8 medium (E8, Gibco) to 80–90% confluency. All cell lines were free of *Mycoplasma sp.* infection, which was tested as previously described^55^.

### Reagents and cellular hypoxia induction

1% O_2_ cellular hypoxia was achieved using a modular hypoxia incubator chamber (Galaxy CO-14S, Eppendorf New Brunswick). To keep hypoxic conditions culture medium was degassed before use and the incubator chamber door was never opened during treatments. MCL-1 inhibitor S63845 (Cayman Chemical) was dissolved in DMSO and stored at − 20 °C protected from light.

### RNA isolation and RT-qPCR

Total RNA was extracted from hPSCs with TRIzol (Thermo Scientific) and cDNA was synthesized from 500 ng of total RNA with 15 mM of random hexamers and MMLV reverse transcriptase (Promega), according to manufacturer's instructions as previously described^5,56^. For qPCR studies, cDNA samples were diluted fivefold and PCR amplification and analysis were performed with StepOnePlus Real-Time PCR System (PE Applied Biosystems). The FastStart Universal SYBR Green Master (Rox) (Roche) was used for all reactions, following the manufacturer´s instructions. For information about primer sequences please see Supplementary methods (Table S1).

### Protein analysis

Protein expression levels were analyzed as previously described^5^. Total proteins were extracted from hPSCs in ice-cold RIPA protein extraction buffer (Sigma) supplemented with protease inhibitors (Protease inhibitor cocktail set I, Calbiochem). Protein concentration was determined using Bicinchoninic Acid Protein Assay (Pierce). Equal amounts of protein were electrophoresed on a 12% SDS–polyacrylamide gel and transferred to PVDF membranes (Millipore). Blots were blocked 1 h at RT (room temperature) in TBS (20 mM Tris–HCl, pH 7.5, 500 mM NaCl) containing low-fat powdered milk (5%) and Tween 20 (0.1%). Incubations with primary antibodies were performed ON (overnight) at 4 °C in blocking buffer (3% skim milk, 0.1% Tween, in Tris-buffered saline). Membranes were then incubated with the corresponding counter-antibody and the proteins were revealed by enhanced chemiluminescence detection (SuperSignal West Femto System, Thermo Scientific). In most cases, full-length blots are not provided in Supplementary information as blots were cut before hybridization with antibodies to save on samples and reagents. For information about the antibodies used please see Supplementary methods (Table S2). Densitometric protein level analysis was performed with ImageJ 1.34 s software (https://imagej.nih.gov/ij/).

### Cell viability assay

hPSCs were plated onto Vitronectin-coated MWx96 cell culture dishes at densities between 3.33 × 10^4^–1 × 10^5^ cells/cm^2^ and grown until confluence as previously described^5,56^. After treatments, 50 μg/well of activated 2,3-bis (2-methoxy-4-nitro-5-sulfophenyl)-5 [(phenylamino) carbonyl]-2 H-tetrazolium hydroxide (XTT) in PBS containing 0.3 μg/well of N-methyl dibenzopyrazine methyl sulfate (PMS) were added (final volume 100 μl) and incubated for 1–2 h at 37 °C. Cellular metabolic activity was determined spectrophotometrically at 450 nm.

### Trypan blue staining

For Trypan blue exclusion assay, hPSCs were seeded on Vitronectin-coated MWx6 tissue culture plates at a density of 1 × 10^5^ cells/cm^2^ and grown to 80–90% confluence. After treatments, adherent and detached cells were collected and stained with 0.4% Trypan blue solution (final concentration 0.08%) for 5 min at room temperature as previously described^5^. Cells were counted in a hemocytometer chamber. Percentages of surviving cells were calculated as the total number of live cells divided by the total number of cells and multiplied by 100.

### Flow cytometric analysis of cell viability by propidium iodide (PI) staining

Single-cell suspensions were obtained with Accutase (37 °C for 7 min). hPSCs were then centrifuged at 200 × g for 5 min and resuspended up to 1 × 10^6^ cells/ml in FACS Buffer (2.5 mM CaCl_2_, 140 mM NaCl and 10 mM HEPES pH 7.4). Next, 100 µl of cellular suspension was incubated with 5 µl of PI (50 µg/ml) in PBS for 5 min in the dark. Finally, 400 µl of FACS Buffer was added to each tube, and cells were immediately analyzed by flow cytometry^5^. Data were acquired on a BD Accuri C6 flow cytometer and analyzed using BD Accuri C6 software.

### DAPI staining

hPSCs were grown on Vitronectin-coated MWx24 cell culture dishes (1 × 10^5^ cells/cm^2^ seeding density) with E8 medium to 80–90% confluency and, after treatments, rinsed with ice-cold PBS and fixed in PBSA (PBS with 0.1% bovine serum albumin) with 4% formaldehyde for 45 min. After two washes cells were permeabilized with 0.1% Triton X-100 in PBSA with 10% normal goat serum for 30 min, washed twice, and stained with 4–6-Diamidino-2-phenylindole (DAPI, Thermo Scientific) for 20 min. Stained cells were examined under a Nikon Eclipse TE2000-S inverted microscope equipped with a 20X E-Plan objective and a super high-pressure mercury lamp. The images were acquired with a Nikon DXN1200F digital camera controlled by the EclipseNet software (version 1.20.0 build 61). Percentages of apoptotic nuclei were calculated as the total number of cells showing chromatin condensation divided by the total number of cells and multiplied by 100^5^.

### Assessment of DNA fragmentation

Apoptosis induction was quantified by direct determination of nucleosomal DNA fragmentation with Cell Death Detection ELISAPlus kit (Roche) as previously described^56^. Briefly, 1 × 10^5^ cells/cm^2^ hPSCs were plated on MWx24 cell culture dishes in 500 μl E8 media and grown to 80–90% confluence. After treatments, cells were lysed according to the manufacturer's instructions, followed by centrifugation (200 × g, 5 min). The mono and oligonucleosomes in the supernatants were determined using an anti-histone-biotinylated antibody. The resulting color development was measured at 405 nm wavelength using a multiplate spectrophotometer. Results were expressed as DNA oligomer fold induction versus normoxia, calculated from the ratio of absorbance of treated samples to that of untreated ones.

### Fluorometric CASPASE-3 activity assay

CASPASE-3 activity was measured with EnzChek® CASPASE-3 Assay Kit #2 (Molecular Probes Inc.) using rhodamine 110 bis-(N-CBZ-L-aspartyl-L-glutamyl-L-valyl-L-aspartic acid amide) (Z-DEVD–R110), according to manufacturer instructions. Briefly, total cell lysates (after counting total cell number with a Neubauer chamber) were prepared after 8, 16, and 24 h of 1% O_2_ hypoxia induction. Cell lysates samples were mixed in MWx96 plates with reaction buffer, and further incubated for 30 min at room temperature avoiding direct light. Then, Z-DEVD-R110 (25 µM) was added to each well and incubated for 1 h at 37 °C in the dark. CASPASE-3 activity of cell extracts was determined by a fluorescence microplate reader (Fluoroskan Ascent FL, Thermo Fisher) using 496/520 nm excitation/emission wavelengths. Blanks were substrate and values were normalized to the number of cells. Results were expressed as CASPASE-3 activity fold induction versus normoxia.

### Immunostaining and fluorescence microscopy

hPSCs were analyzed for in situ immunofluorescence^5,56^. Briefly, cells were rinsed with ice-cold PBS and fixed in PBSA (PBS with 0.1% bovine serum albumin) with 4% formaldehyde for 45 min. After two washes cells were permeabilized with 0.1% Triton X-100 in PBSA with 10% normal goat serum for 30 min, washed twice, and stained with a rabbit polyclonal antibody anti-active CASPASE-3 (ab13847, Abcam Inc., Cambridge, MA, USA). Fluorescent secondary antibody Alexa Fluor 488-conjugated anti-rabbit IgG (Thermo Scientific) was used to localize the antigen/primary antibody complexes. Cells were counterstained with DAPI and examined under a Nikon Eclipse TE2000-S inverted microscope equipped with a 20X E-Plan objective and a super high-pressure mercury lamp. The images were acquired with a Nikon DXN1200F digital camera controlled by the EclipseNet software (version 1.20.0 build 61).

### Cell transfection and RNA Interference

Cells were transfected with the corresponding small interfering RNA (siRNA) using Lipofectamine™ RNAiMAX lipid reagent (Thermo Scientific) as per manufacturer's instructions and previously described^5,56^. Briefly, 1 × 10^5^ cells/cm^2^ hPSCs were plated unicellular on Vitronectin-coated MWx24 cell culture dishes, grown 24 h with E8 media, and then transfected with Silencer Select Negative Control #2 (Ambion™, Cat. #4,390,846) or Silencer Select Validated HIF-1α siRNA (Ambion™, Cat. #4,390,824, siRNA ID: s6539) or Silencer Validated P53 siRNA (Ambion™, Cat. # AM51331, siRNA ID: 106,141). The concentration of siRNA used for cell transfection was 20 nM for HIF-1α siRNA and 50 nM for P53 siRNA.

### Statistical analysis

All results are expressed as mean ± SEM. One-way ANOVAs followed by Dunnett's multiple comparisons tests or two-tailed Student´s t-test were used to detect significant differences (*p* < 0.05) among treatments as indicated.

## Supplementary Information

Supplementary Information.

## Data Availability

The datasets used and/or analyzed during the current study are available from the corresponding author on reasonable request.
