# Supplementary material for: Acute severe hypoxia induces apoptosis of human pluripotent stem cells by a HIF-1α and P53 independent mechanism
Source: Sci Rep. 2022 Nov 5;12:18803. doi: 10.1038/s41598-022-23650-7 (PMC9637190; doi:10.1038/s41598-022-23650-7)
Supplement: Supplementary file 1 — Supplementary Information. [file 41598_2022_23650_MOESM1_ESM.pdf]

Supplementary information

Full Title: *"Acute severe hypoxia induces apoptosis of human pluripotent stem cells by a HIF-1α and P53 independent mechanism"*

Authors: Sofía Mucci[1], Luciana Isaja[1], María Soledad Rodríguez-Varela, Sofía Luján Ferriol-Laffouillere, Mariela Marazita, Guillermo Agustín Videla-Richardson, Gustavo Emilio Sevlever, María Elida Scassa and Leonardo Romorini[*].

Author affiliations: Laboratorios de Investigación Aplicada en Neurociencias (LIAN-CONICET), Fundación para la Lucha contra las Enfermedades Neurológicas de la Infancia (Fleni), Ruta 9, Km 52.5, Belén de Escobar, Provincia de Buenos Aires, B1625XAF, Argentina.

[1] Both authors contributed equally to this work.

[*] Corresponding author: Leonardo Romorini, e-mail: lromorini@fleni.org.ar

Supplementary methods:

Antibodies and primers used:

	Primer sequence (5' → 3')	
**Name**	**Forward**	**Reverse**
RPL7	AATGGCGAGGATGGCAAG	TGACGAAGGCGAAGAAGC
BNIP-3	ATCGCCGCAGTTGCCCTCTGG	ATAGAAACCGAGGCTGGAACGCTG
BNIP-3L	AATGTCGTCCCACCTAGTCG	CCCCCATTTTTCCCATTGCC
VEGF	GTAGCTCGGAGGTCGTGGCG	AATTCCAGCACCGAGCGCCC
MCL-1	GGGCAGGATTGTGACTCTCATT	GATGCAGCTTTCTTGGTTTATGG
BAX	GACGGCAACTTCAACTGG	GTGAGGAGGCTTGAGGAG
BCL-2	TATAACTGGAGAGTGCTGAAG	ACTTGATTCTGGTGTTTCCC
BCL-XL	TGCGTGGAAAGCGTAGACAAG	GTGGGAGGGTAGAGTGGATGG
NOXA	ACCAAGCCGGATTTGCGATT	ACTTGCACTTGTTCCTCGTGG
PUMA	GACCTCAACGCACAGTACGAG	AGGAGTCCCATGATGAGATTGT
P53	CAATAGGTGTGCGTCAGAAG	CTTACATCTCCCAAACATCCC
HIF-1α	ACCCACCGCTGAAACGCCAA	GGTGTCTGATCCTGAATCTGGGGC

Supplementary Table S1. *Primers used for RT-qPCR experiments.*

Antibody	Specie	Brand	N° Catalogue	Dilution
α-HIF-1α	Monoclonal-Mouse	BD	610958	1/1000
α-HIF-2α	Polyclonal-Rabbit	Abcam	ab199	1/1000
α-CASPASE-9	Polyclonal-Rabbit	Cell Signaling	9502	1/1000

α-active CASPASE-3	Polyclonal-Rabbit	Abcam	ab13847	1/1000
α-PARP	Monoclonal-Mouse	Santa Cruz	sc-8007	1/1000
α-BAX	Polyclonal-Rabbit	Santa Cruz	sc-493	1/1000
α-MCL-1	Monoclonal-Rabbit	Cell Signaling	94296	1/1000
α-BCL-2	Monoclonal-Mouse	Santa Cruz	sc-7382	1/1000
α-BCL-X_L	Polyclonal-Rabbit	Santa Cruz	sc-634	1/1000
α-PUMA	Monoclonal-Rabbit	Abcam	ab33906	1/1000
α-NOXA	Monoclonal-Rabbit	Cell Signaling	14766	1/1000
α-P53	Monoclonal-Mouse	Abcam	ab1101	1/1000
α-ACTIN	Polyclonal-Goat	Santa Cruz	sc-1616	1/1000

Supplementary Table S2. *Primary antibodies used for western blot and immunofluorescence experiments.*

Supplementary figures:

Graphs and statistical analysis of Figure 2c Propidium iodide (PI) histograms

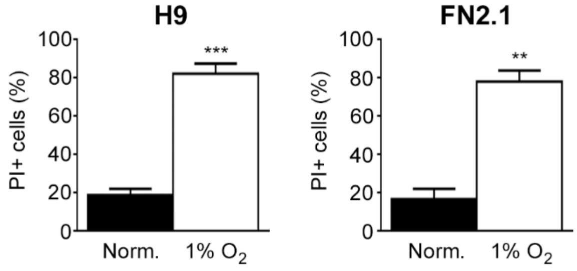

Supplementary Figure S1. ***Graphs and statistical analysis of Figure 2c Propidium iodide (PI) histograms. The percentage*** of PI-positive cells (late apoptotic or necrotic) was determined by flow cytometric analysis upon 1% O_2 (24 hours) hypoxia treatment. Mean + SEM from three independent experiments is graphed. Norm.: normoxia. Statistical analysis was done by Student's t-test, (***) p<0.001 and (**) p<0.01 vs. normoxia.

Quantification of Figure 3a western blot images

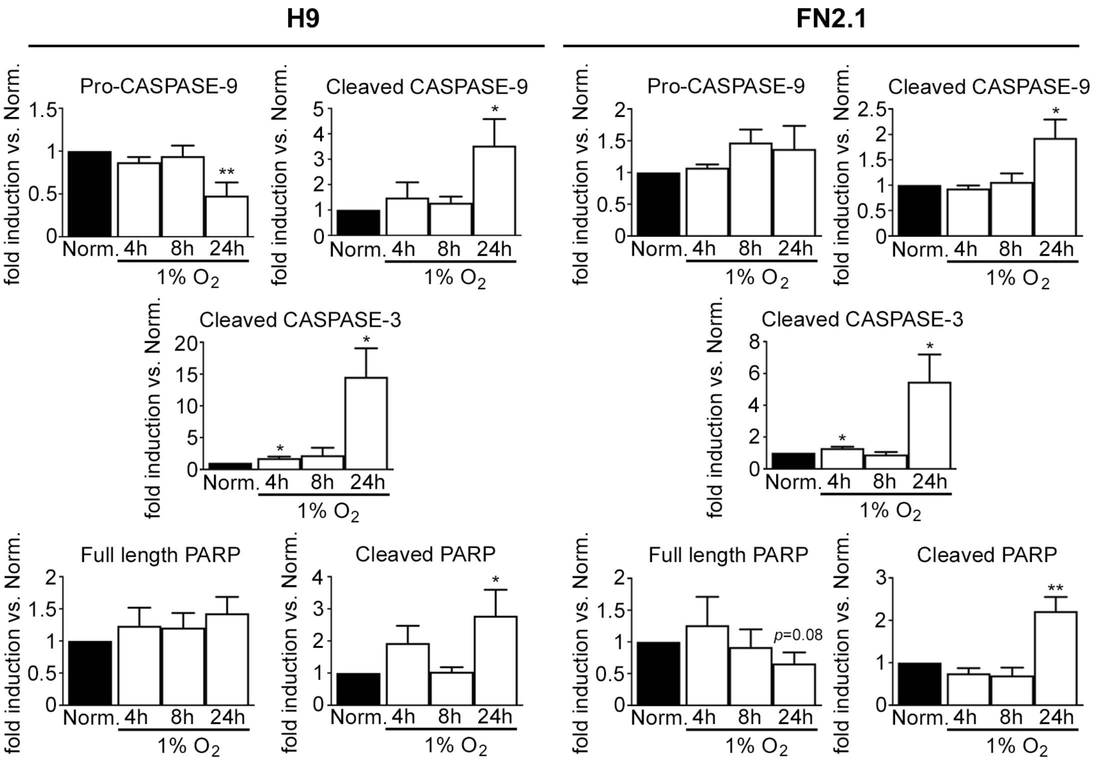

Supplementary Figure S2. ***Quantification of Figure 3a western blot images.*** Bar graphs represent the densitometric quantification of bands from western blot images shown in Figure 3a. Norm.: normoxia. Data are expressed as means + SEM fold induction relative to normoxia (arbitrarily set as 1) and Statistical analysis was done by Student's t-test, (**) p<0.01 and (*) p<0.05 vs. normoxia.

CASPASE-3 activation by immunofluorescence upon 1% O_2 hypoxia induction

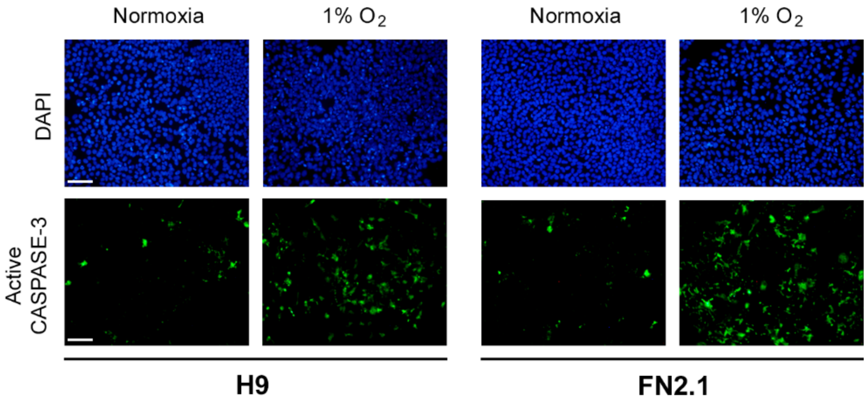

Supplementary Figure S3. ***CASPASE-3 activation upon 1% O_2 hypoxia induction.*** Representative micrographs of H9 and FN2.1 hPSCs immunostained against active CASPASE-3 (green) after 24 hours of treatment with 1% O_2. The nuclei were counterstained with DAPI. Scale bars represent 100 μm.

Quantification of Figure 4b western blot images

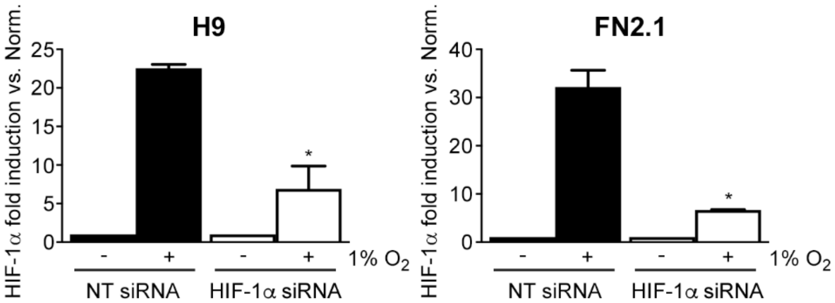

Supplementary Figure S4. ***Quantification of Figure 4b western blot images.*** Bar graphs represent the densitometric quantification of bands from western blot images (n=3) shown in Figure 4b. Norm.: normoxia. Data are expressed as

means + SEM fold induction relative to normoxia (arbitrarily set as 1) and Statistical analysis was done by Student's t-test, (*) $p<0.05$ vs. NT siRNA + 1% O_2.

Quantification of Figure 5a western blot images

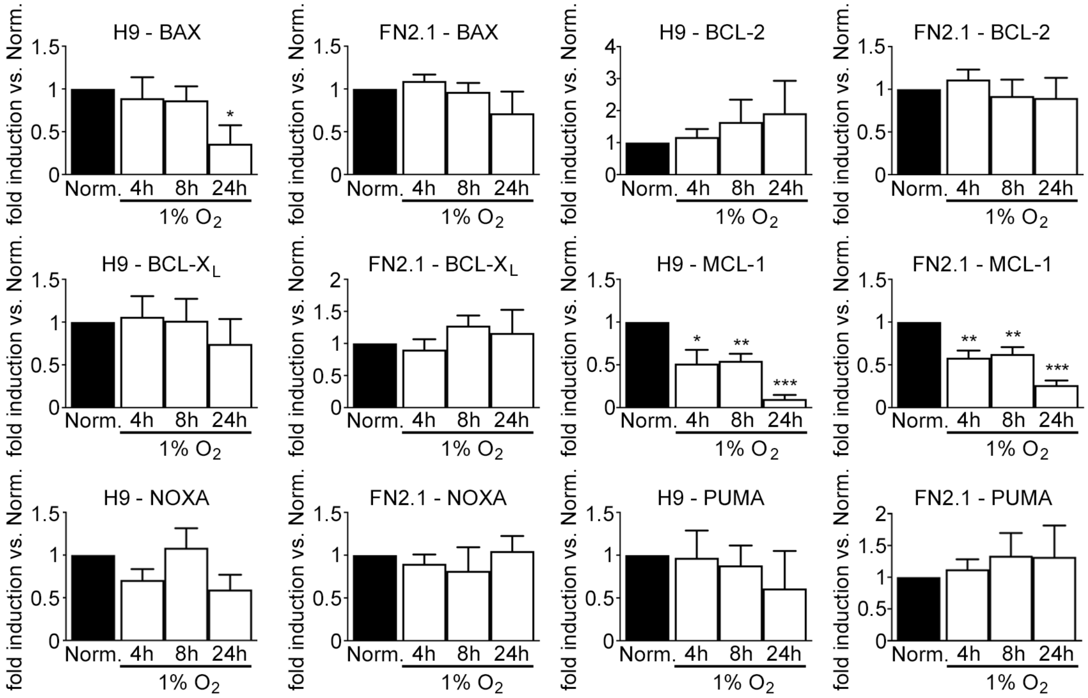

Supplementary Figure S5. ***Quantification of Figure 5a western blot images.*** Bar graphs represent the densitometric quantification of bands from western blot images shown in Figure 5a. Norm.: normoxia. Data are expressed as means + SEM fold induction relative to normoxia (arbitrarily set as 1) and Statistical analysis was done by Student's t-test, (*) $p<0.05$, (**) $p<0.01$, and (***) $p<0.001$ vs. normoxia.

BAX, BCL-2, BCL-XL, NOXA, and PUMA mRNA expression levels analysis

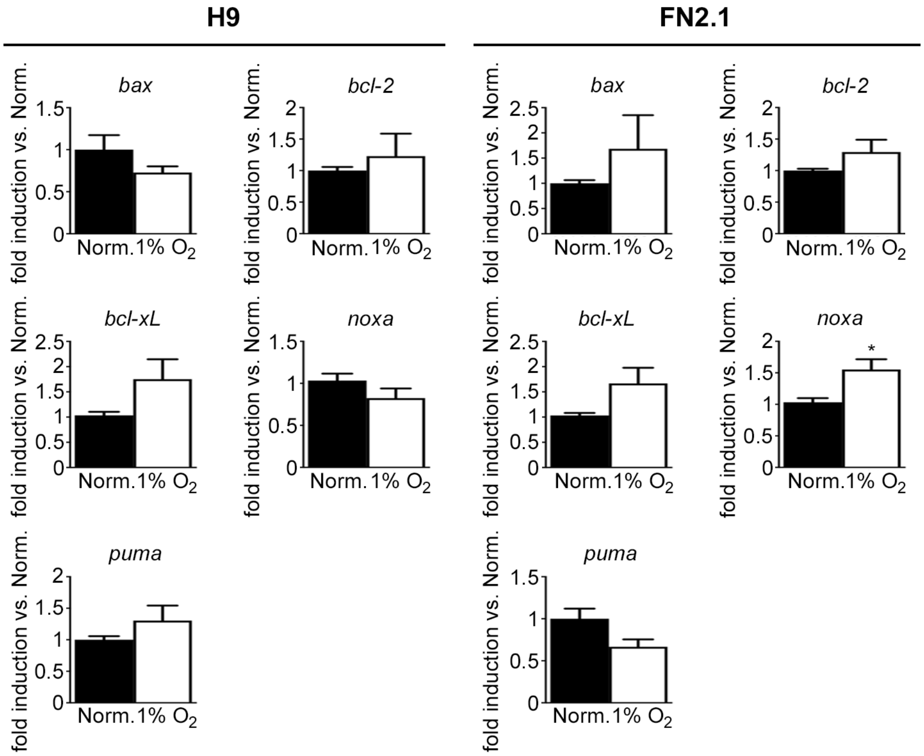

Supplementary Figure S6. ***BAX, BCL-2, BCL-XL, NOXA, and PUMA mRNA expression levels analysis.*** mRNA expression levels of *BAX, BCL-2, BCL-XL, NOXA,* and *PUMA* were analyzed by RT-qPCR upon 1% O_2 (24 hours) hypoxia induction. *RPL7* expression was used as normalizer. Norm.: normoxia. Graph shows mean + SEM mRNA fold induction relative to normoxia (arbitrarily set as 1) from three independent experiments. Statistical analysis was done by Student's t-test, (*) $p<0.05$ vs. normoxia.

Effect of siRNA-mediated down regulation of P53 in hPSCs viability and death upon 1% O_2 treatment

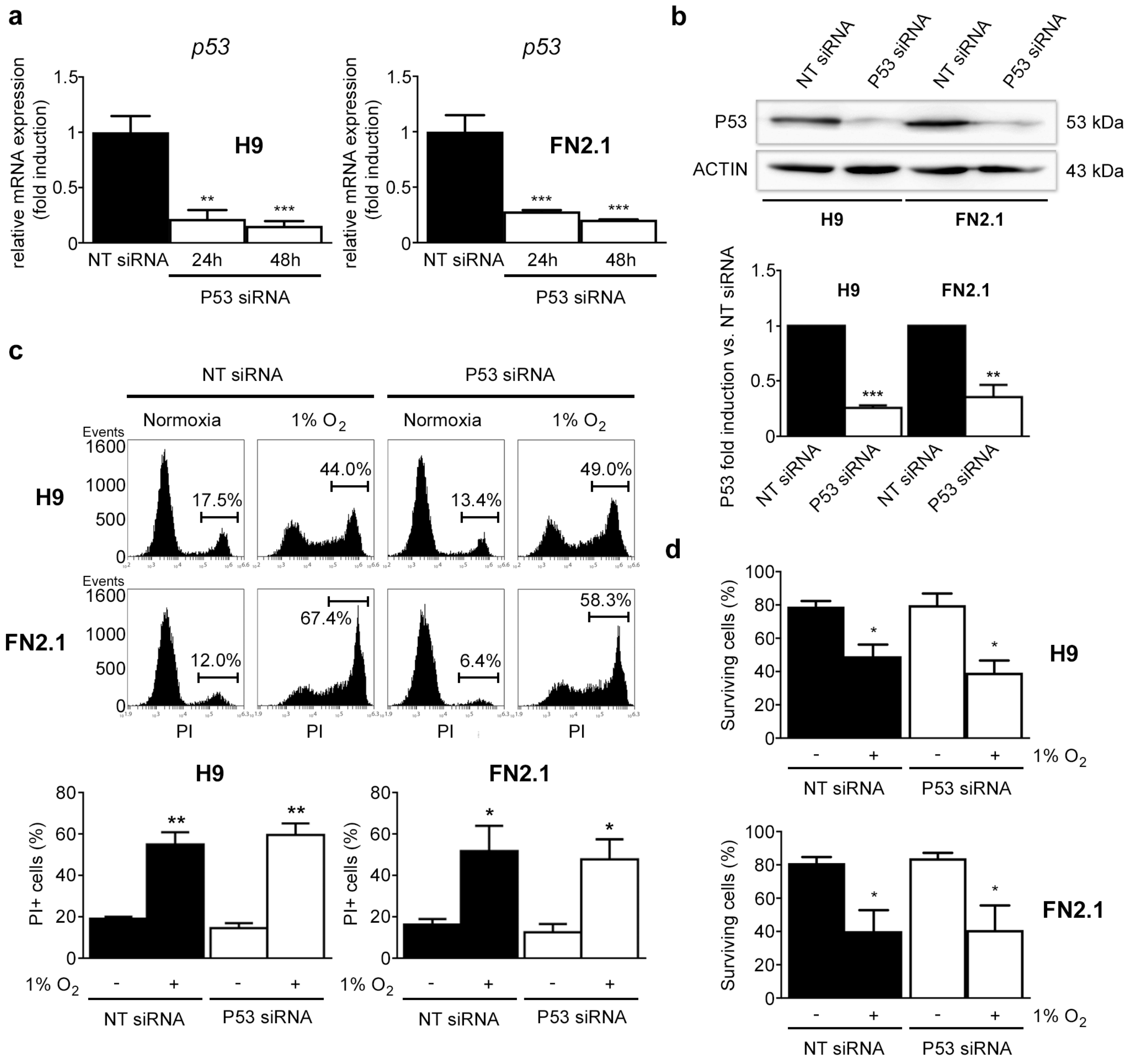

Supplementary Figure S7. ***Effect of siRNA-mediated down regulation of P53 in hPSCs viability and death upon 1% O_2 treatment***. H9 hESCs and FN2.1 hiPSCs were transfected with negative control non-targeting siRNA (NT siRNA) (50 nM) or P53 siRNA (50 nM) and then: **(a)** mRNA expression levels of *P53* were analyzed by RT-qPCR at 24 and 48 hours (h) post siRNAs transfection. *RPL7* mRNA expression levels were used as normalizer. Graph shows mean + SEM mRNA fold induction relative to NT siRNA transfectants arbitrarily set as 1 from three independent experiments. Statistical analysis was done by Student's t-test, (**) $p<0.01$ and (***) $p<0.001$ vs. NT

siRNA. **(b)** Expression levels of P53 were analyzed by western blot in H9 and FN2.1 cells at 48 hours post siRNAs transfection. ACTIN was used as loading control. Representative blots of three independent experiments are shown (original western blot images are presented in Supplementary Fig. S16). Bar graphs represent the densitometric quantification of bands from western blot images. Data are expressed as mean+ SEM fold induction relative to NT siRNA treatment (arbitrarily set as 1) (n=3) and Statistical analysis was done by Student's t-test, (**) p<0.01 and (***) p<0.001 vs. NT siRNA. **(c)** Representative histograms of Propidium iodide (PI) stained H9 and FN2.1 unfixed cells at 48 h post siRNA transfection. 1% O_2 hypoxia was induced at 24 h post siRNA transfection. The percentage of PI-positive cells (late apoptotic or necrotic) was determined by flow cytometric analysis. Mean + SEM from three independent experiments is graphed. Statistical analysis was done by Student's t-test, (**) p<0.01 and (*) p<0.05 vs. normoxia. **(d)** Bar graphs show the percentage of surviving cells assessed by Trypan blue exclusion method at 48 h post siRNA transfection. 24 h after transfection cells were incubated in 1% O_2. Mean + SEM from three independent experiments is shown. Statistical analysis was done by Student's t-test, (*) p<0.05 vs. NT siRNA.

Full-length and original western blot images of Figure 1

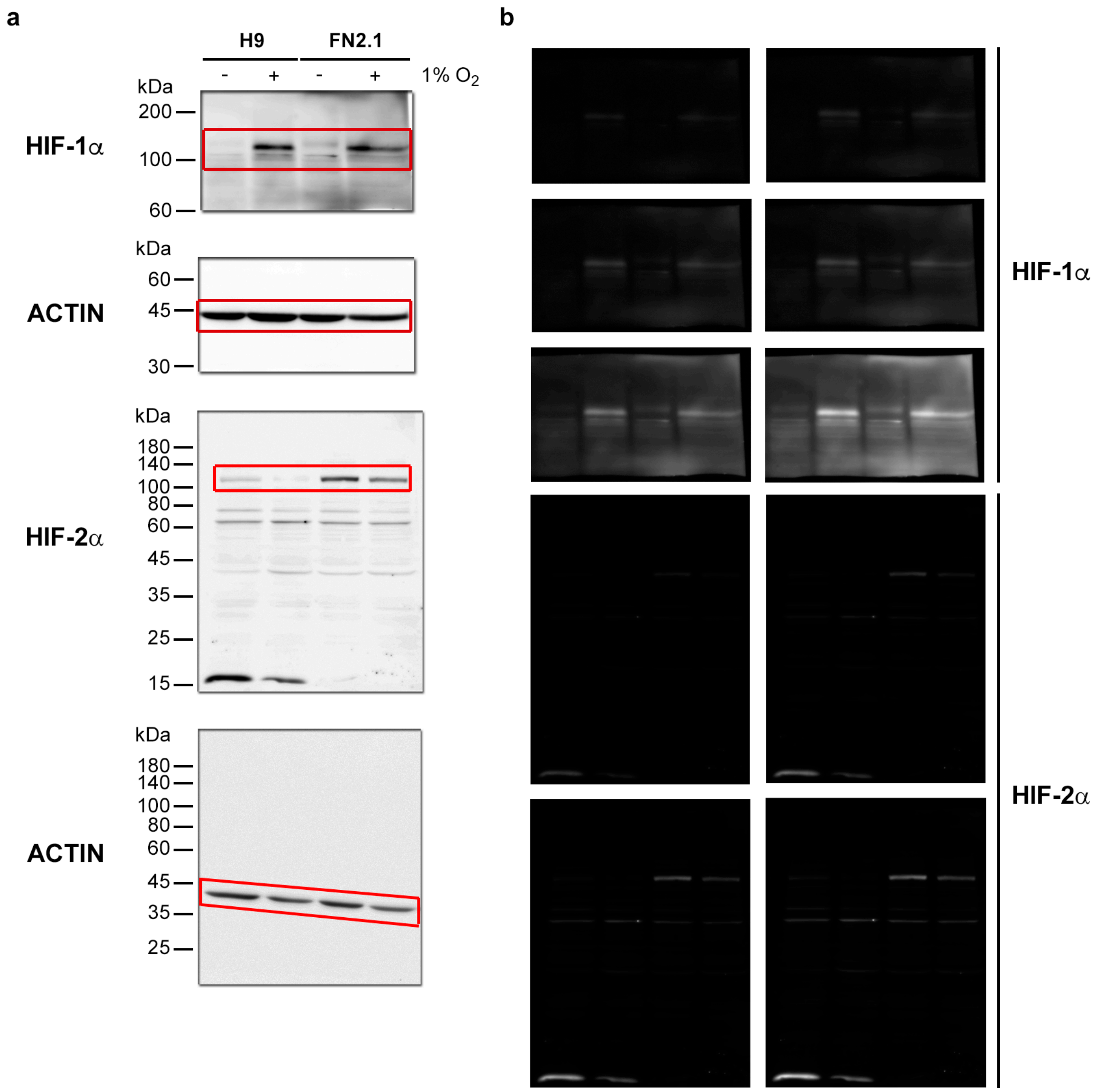

Supplementary Figure S8. Full-length *and original western blot images of Figure 1: HIF-1α and HIF-2α protein expression levels upon 1% O_2 incubation.* *HIF-1α* and *HIF-2α* protein expression levels were analyzed by western blot in H9 hESCs and FN2.1 hiPSCs upon 1% O_2 (24 hours) treatment. ACTIN was used as loading control. Some blots were cut before hybridization with antibodies to optimize the use of the samples. **(a)** Original (HIF-1α) and full-length (HIF-2α) blot images are shown in main Figure 1. Regions used in the main figure are denoted using red boxes. **(b)** Multiple inverted exposure images (with visible membrane edges) of HIF-1α and HIF-2α original blots used in

the main figure. ColorBurst Electrophoresis Marker from Sigma (C1992) and Blue Plus IV Protein Marker from TransGen Biotech (DM131) were used.

Replicates of western blot images of Figure 1

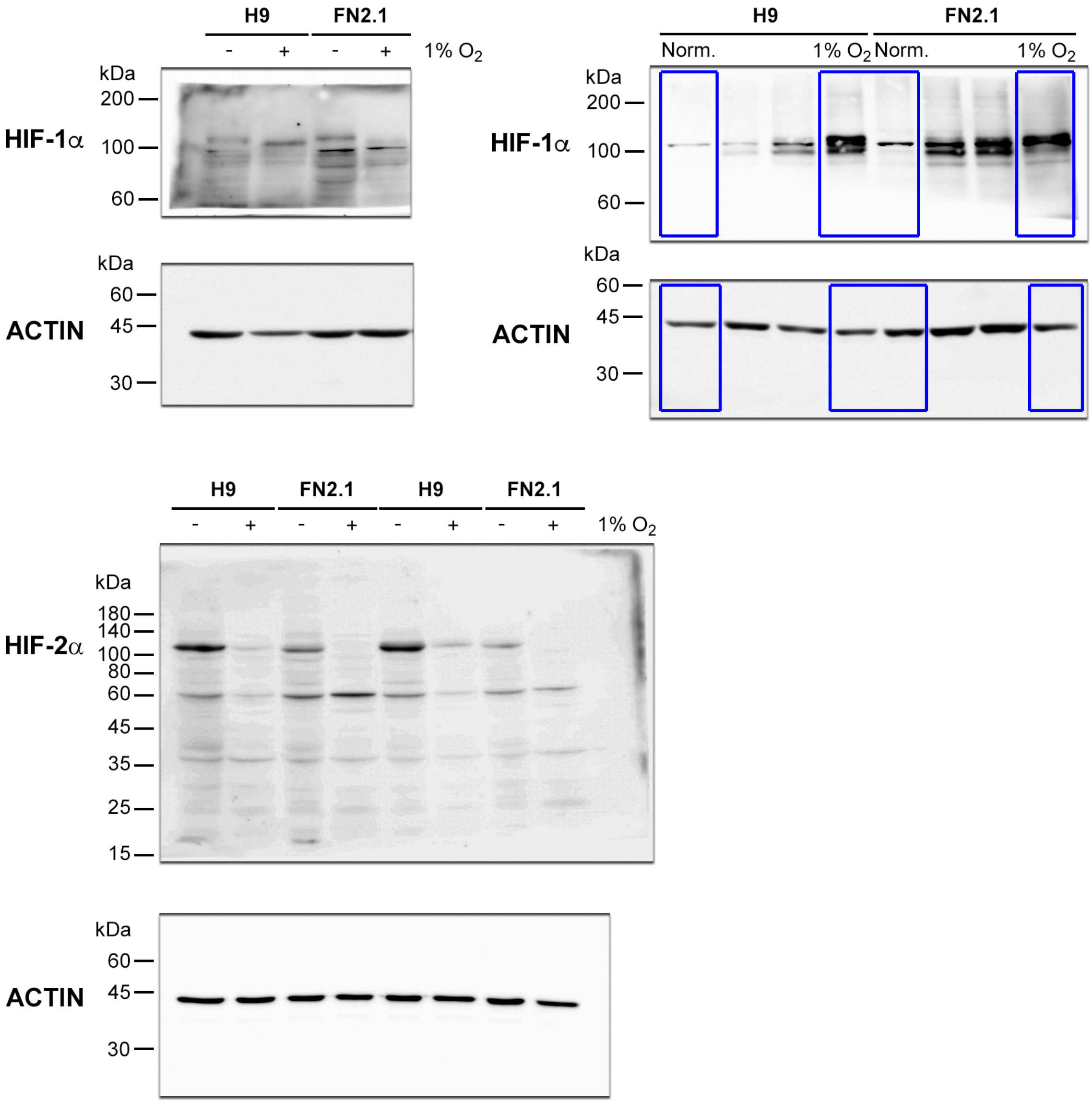

Supplementary Figure S9. ***Replicates of western blot images of Figure 1: HIF-1α and HIF-2α protein expression levels upon 1% O_2 incubation.*** *HIF-1α* and *HIF-2α* protein expression levels were analyzed by western blot in H9 hESCs and FN2.1 hiPSCs upon 1% O_2 (24 hours) treatment. ACTIN was used as loading control. Some blots were cut before hybridization with antibodies to optimize the use of the samples. Blot images of all replicates performed

for HIF-1α, HIF-2α, and their respective loading controls (ACTIN) are shown. Regions denoted using blue boxes are those that refer to the treatments of interest. ColorBurst Electrophoresis Marker from Sigma (C1992) and Blue Plus IV Protein Marker from TransGen Biotech (DM131) were used.

Original western blot images of Figure 3

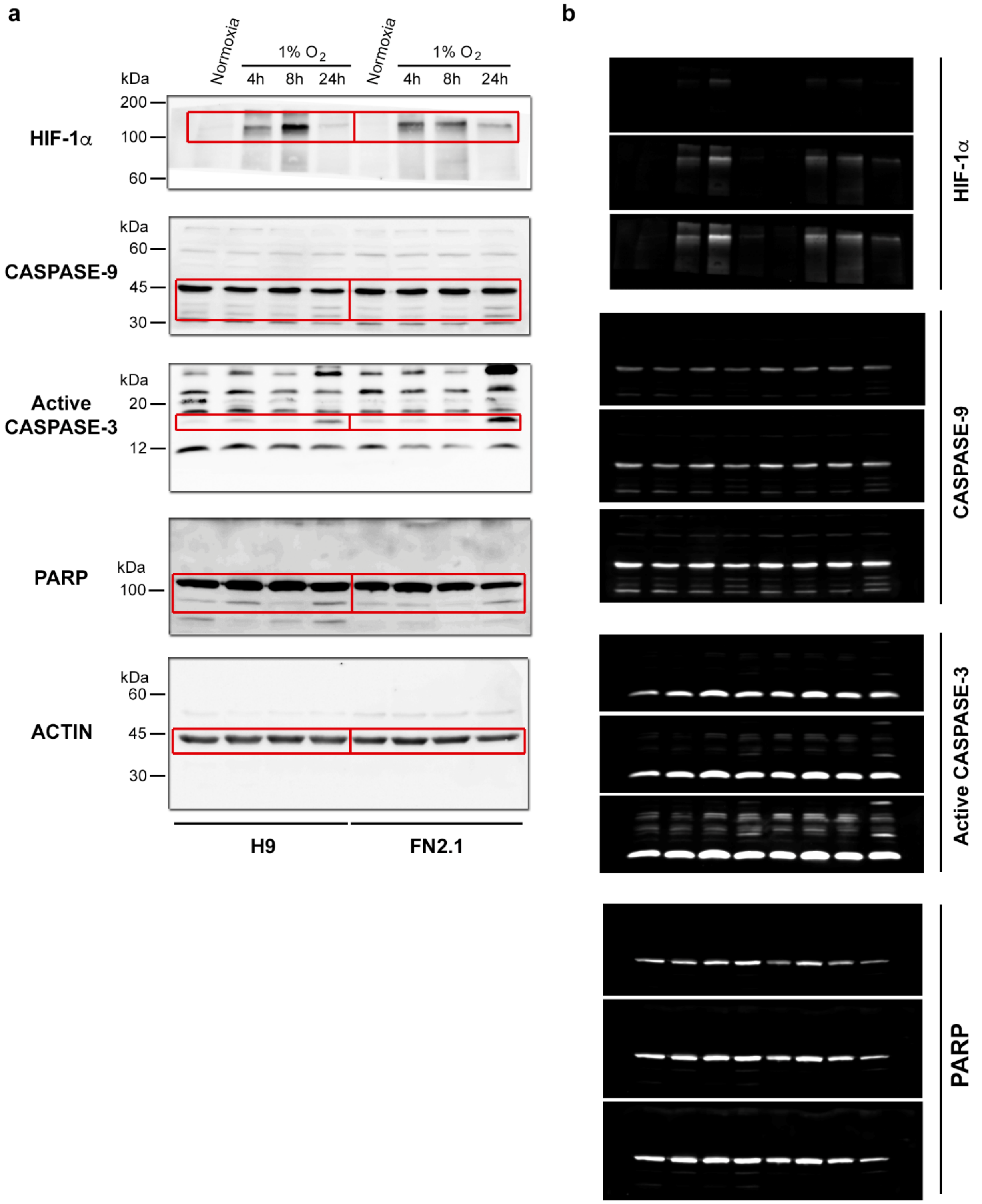

Supplementary Figure S10. ***Original western blot images of Figure 3: CASPASE-9, CASPASE-3 activation, and PARP cleavage upon 1% O_2 hypoxia induction.*** Cleavage and activation of initiator CASPASE-9, effector

CASPASE-3, PARP proteolysis (CASPASE-3 substrate), and HIF-1α stabilization were analyzed by western blot in H9 hESCs and FN2.1 hiPSCs at 4-, 8- and 24-hours post 1% O_2 treatment. ACTIN was used as loading control. Blots were cut before hybridization with antibodies to optimize the use of the samples. **(a)** Original Figure 3 blots images are shown. Regions used in the main figure are denoted using red boxes. **(b)** Multiple exposure-time inverted images (with visible membrane edges) of some of the original blots used in the main figure. In all cases, ColorBurst Electrophoresis Marker from Sigma (C1992) was used.

Replicates of western blot images of Figure 3

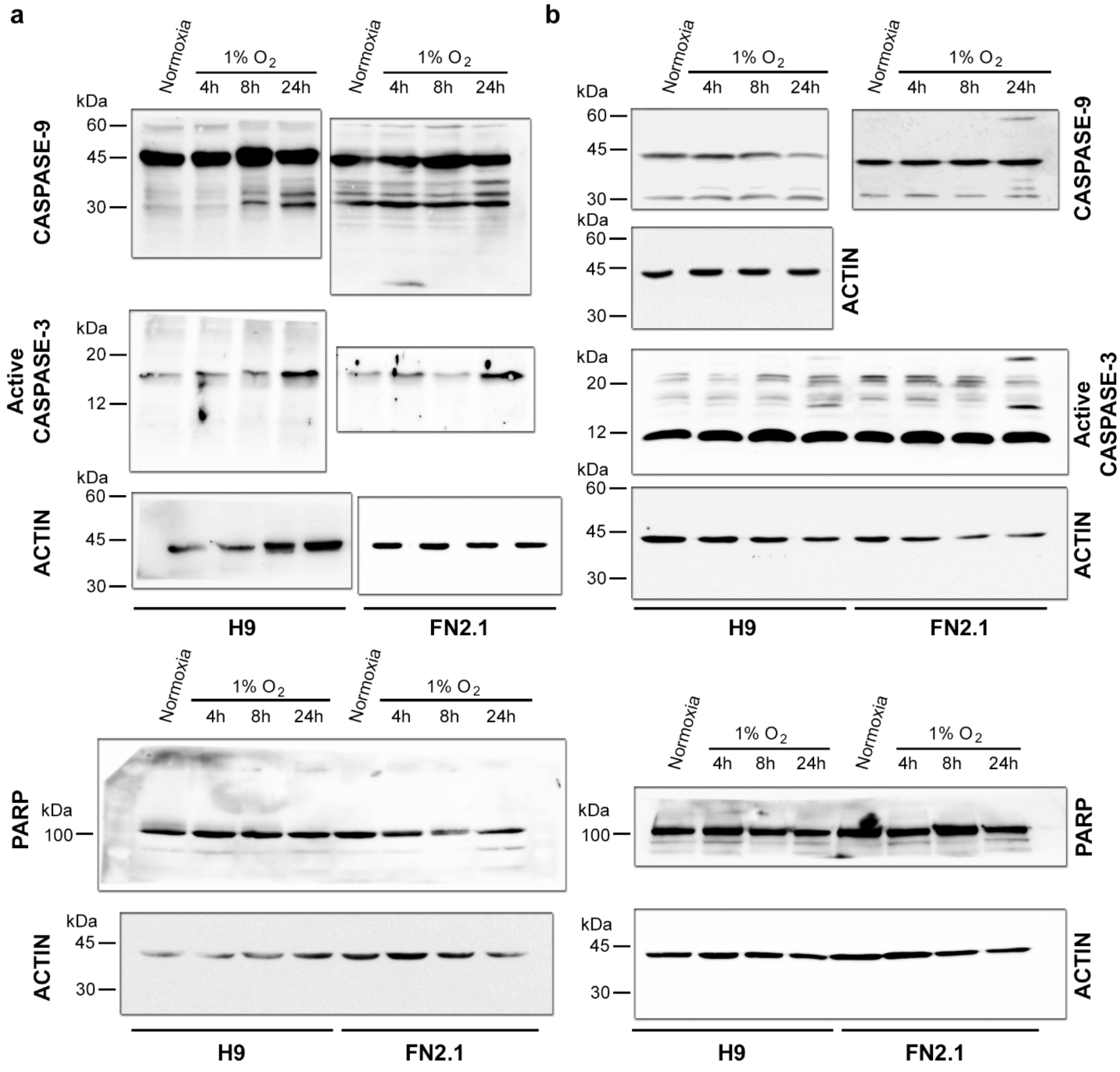

Supplementary Figure S11. ***Replicates of western blot images of Figure 3: CASPASE-9, CASPASE-3 activation, and PARP cleavage upon 1% O_2 hypoxia induction.*** Cleavage and activation of initiator CASPASE-9, effector CASPASE-3, and PARP proteolysis (CASPASE-3 substrate) were analyzed by western blot in H9 hESCs and FN2.1 hiPSCs at 4-, 8- and 24-hours post 1% O_2 treatment. ACTIN was used as loading control. Blots were cut before hybridization with antibodies to optimize the use of the samples. Blot images of duplicates **(a)** and triplicates **(b)** with their respective loading controls (ACTIN) are shown. In all cases, ColorBurst Electrophoresis Marker from Sigma (C1992) was used.

Full western blot images of Figure 4

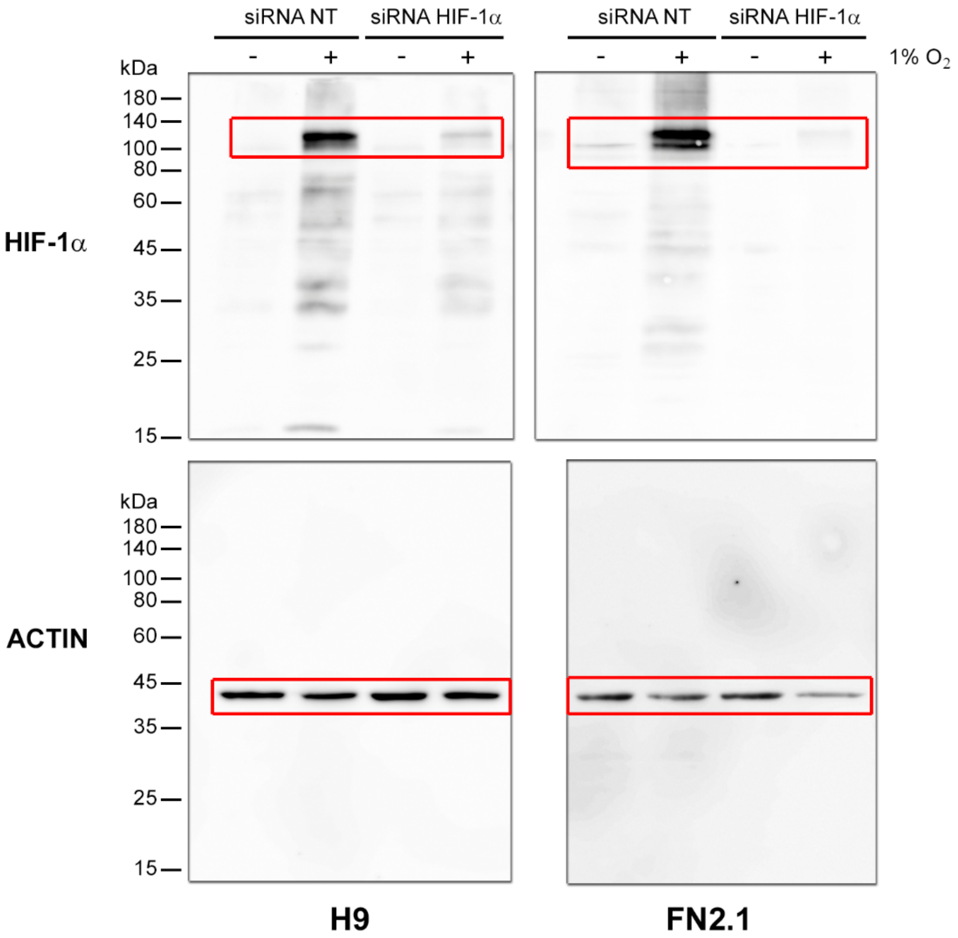

Supplementary Figure S12. ***Full western blot images of Figure 4:*** H9 hESCs and FN2.1 hiPSCs were transfected with negative control non-targeting siRNA (NT siRNA) (20nM) or HIF-1α siRNA (20nM) and then expression levels of HIF-1α were analyzed by western blot in H9 and FN2.1 cells at 48 hours post siRNAs transfection. HIF-1α was stabilized by hypoxia (1% O_2 for 24 hours starting at 24 hours post siRNAs transfection) treatment. ACTIN was used as loading control. Original Figure 4 full-length blots images are shown. Regions used in the main figure are denoted using red boxes. In all cases, Blue Plus IV Protein Marker from TransGen Biotech (DM131) was used.

Original western blot images of Figure 5

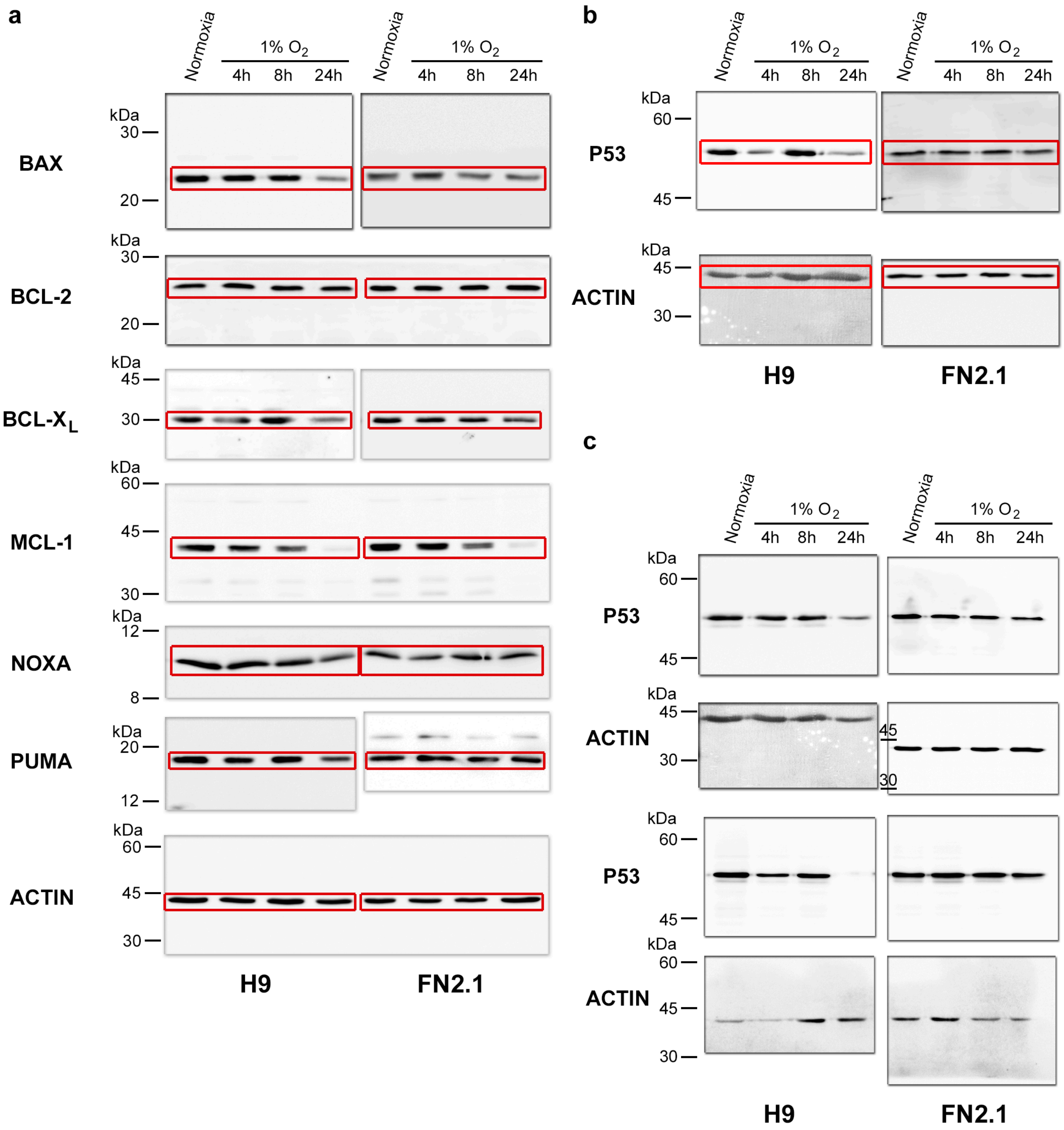

Supplementary Figure S13. ***Original western blot images of Figure 5: BCL-2 family members and P53 protein expression levels.*** Expression levels of **(a)** BCL-2 family members, including BCL-XL (anti-apoptotic), BCL-2 (anti-apoptotic), MCL-1 (anti-apoptotic), BAX (pro-apoptotic), NOXA (pro-apoptotic), and PUMA (pro-apoptotic) or **(b)** P53 were analyzed by western blot in H9 and FN2.1 cells at 4, 8 and 24 hours upon 1% O_2 hypoxia treatment. ACTIN

was used as loading control. Blots were cut before hybridization with antibodies to optimize the use of the samples. Original Figure 5 blots images are shown. Regions used in the main figure are denoted using red boxes. **(c)** Blot images of P53 replicate images with their respective loading controls (ACTIN) are shown. Some blots in Supplementary figures 12, 13, and 14 shared the same ACTIN loading control. In all cases, ColorBurst Electrophoresis Marker from Sigma (C1992) was used.

Replicates of western blot images of Figure 5

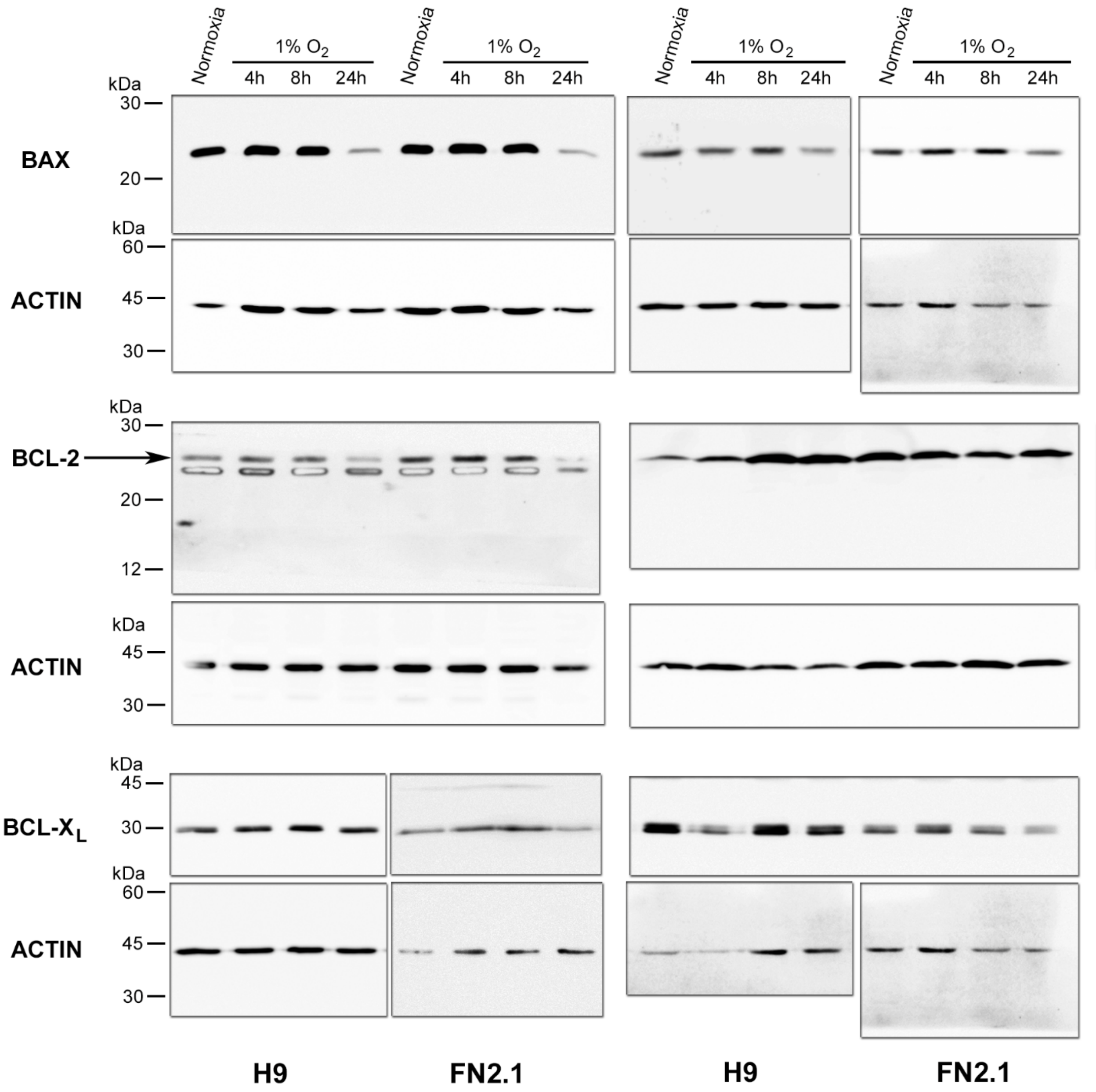

Supplementary Figure S14. ***Replicates of western blot images of Figure 5: BCL-2 family members and P53 protein expression levels.*** Expression levels of BAX, BCL-2, and BCL-XL were analyzed by western blot in H9 and FN2.1 cells at 4, 8, and 24 hours upon 1% O_2 hypoxia treatment. ACTIN was used as loading control. Blots were cut before hybridization with antibodies to optimize the use of the samples. Blot images of BAX, BCL-2 and BCL-XL replicate images with their respective loading controls (ACTIN) are shown. Some blots in Supplementary figures 12, 13, and

14 shared the same ACTIN loading control. In all cases, ColorBurst Electrophoresis Marker from Sigma (C1992) was used.

More replicates of western blot images of Figure 5

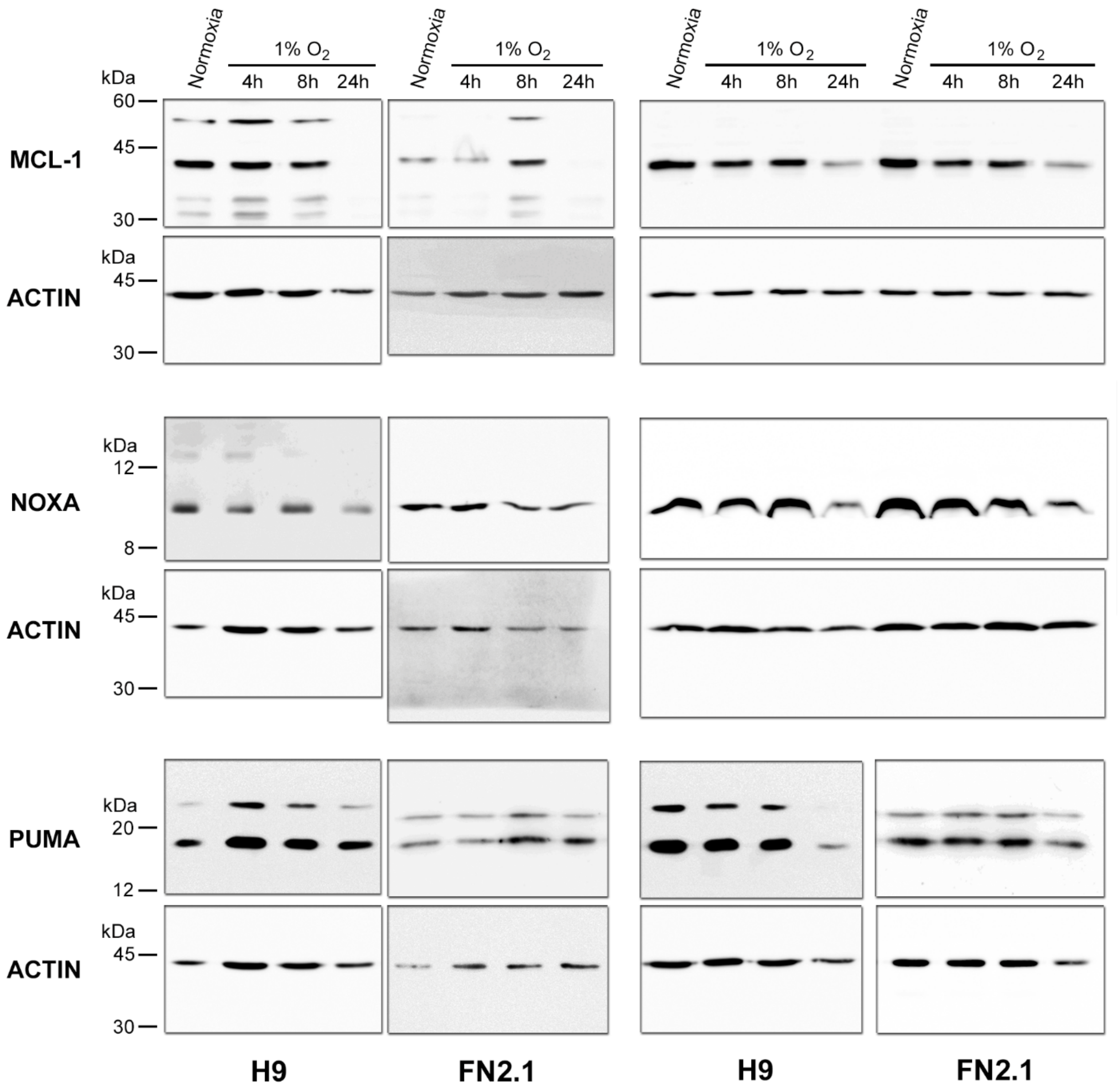

Supplementary Figure S15. ***More replicates of western blot images of Figure 5: BCL-2 family members and P53 protein expression levels.*** Expression levels of MCL-1, NOXA, and PUMA were analyzed by western blot in H9 and FN2.1 cells at 4, 8, and 24 hours upon 1% O_2 hypoxia treatment. ACTIN was used as loading control. Blots were cut before hybridization with antibodies to optimize the use of the samples. Blot images of MCL-1, NOXA,

and PUMA replicate images with their respective loading controls (ACTIN) are shown. Some blots in Supplementary figures 12, 13, and 14 shared the same ACTIN loading control. In all cases, ColorBurst Electrophoresis Marker from Sigma (C1992) was used.

Original western blot images of Supplementary Figure S7

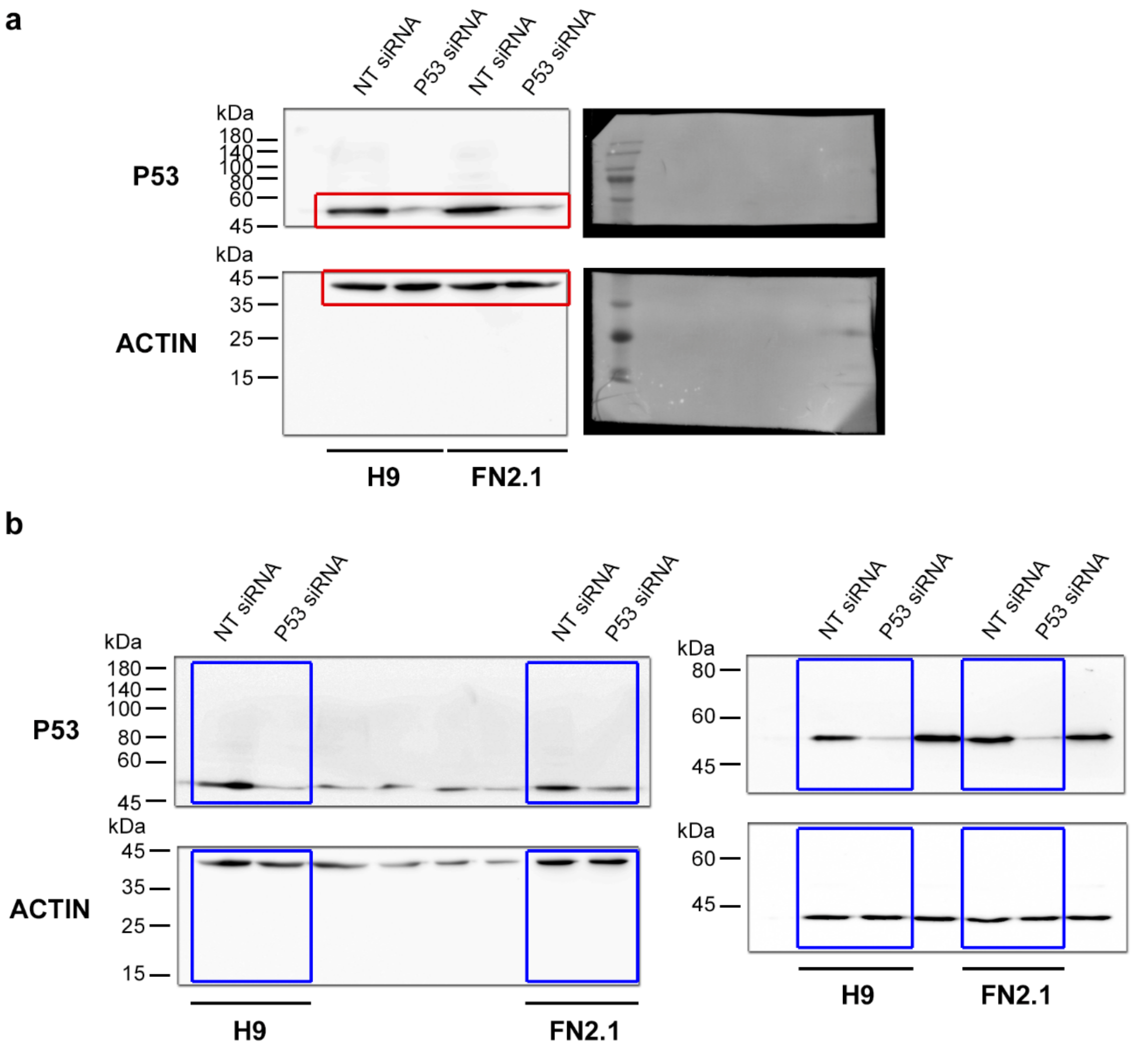

Supplementary Figure S16. ***Original western blot images of Supplementary Figure S7: Effect of siRNA-mediated down regulation of P53 in hPSC viability and death upon 1% O_2 treatment.*** H9 hESCs and FN2.1 hiPSCs were transfected with negative control non-targeting siRNA (NT siRNA) (50nM) or P53 siRNA (50nM) and then expression levels of P53 were analyzed by western blot in H9 and FN2.1 cells at 48 hours post siRNAs transfection.

ACTIN was used as loading control. Blots were cut before hybridization with antibodies to optimize the use of the samples. **(a)** Original Supplementary Figure S7 blots images are shown. Regions used in the main figure are denoted using red boxes. Membranes with visible edges are shown. **(c)** Blot images of all replicates performed. Regions denoted using blue boxes are those that refer to the treatments of interest. In all cases, Blue Plus IV Protein Marker from TransGen Biotech (DM131) was used.
